# Screening of Four Microbes for Solid-State Fermentation of Hawk Tea to Improve Its Flavor: Electronic Nose/GC-MS/GC-IMS-Guided Selection

**DOI:** 10.3390/foods15020324

**Published:** 2026-01-15

**Authors:** Yi-Ran Yang, Wei-Guo Cao, Chen-Yu Li, Shu-Yan Li, Qin Huang

**Affiliations:** 1 School of Chinese Materia Medica, Chongqing University of Chinese Medicine, Chongqing 402760, China; yyiran1127@163.com (Y.-R.Y.); caoweiguo@cqctcm.edu.cn (W.-G.C.); 19823906106@163.com (C.-Y.L.); lishuyan032@163.com (S.-Y.L.); 2 College of Traditional Chinese Medicine, Chongqing Medical University, Chongqing 400016, China

**Keywords:** hawk tea, fermentation, ultra-fast GC e-nose, GC-MS, GC-IMS, OAV, ROAV

## Abstract

Hawk tea (*Litsea coreana* Levl. var. *lanuginosa*), a naturally caffeine-free herbal beverage widely consumed in Southwest China, is characterized by a pronounced camphoraceous note that often deters first-time consumers. In this study, hawk tea leaves were subjected to solid-state fermentation with four microbial strains—*Monascus purpureus*, *Aspergillus cristatus*, *Bacillus subtilis*, and *Blastobotrys adeninivorans*. The volatile compounds of unfermented and fermented hawk teas were identified by ultra-fast gas chromatography electronic nose (ultra-fast GC e-nose), gas chromatography–mass spectrometry (GC-MS) and gas chromatography–ion mobility spectrometry (GC-IMS) analyses, respectively. Furthermore, the calculation of odor activity values (OAVs) and relative odor activity value (ROAV) revealed that 6 and 25 volatile chemicals, including perillaldehyde (OAV 3.692) and linalool (ROAV 100), were the main contributors to the floral, fruity, and woody aroma of fermented hawk tea. Sensory evaluation confirmed that fermentation generally enhanced woody notes while significantly reducing the characteristic camphoraceous and oil oxidation odors. Notably, the *Blastobotrys adeninivorans*-fermented sample exhibited the most pronounced floral and fruity nuances, accompanied by significantly elevated aroma complexity and acceptability. Consequently, *Blastobotrys adeninivorans* represents a promising starter culture for the improvement of hawk tea flavor.

## 1. Introduction

Hawk tea (*Litsea coreana* Levl. var. *lanuginosa*) is a traditional non-caffeinated herbal tea with a long history of consumption in southwestern China, particularly in Chongqing [1,2]. It is characterized by its golden-yellow infusion with reddish hues, a fragrant aroma, and a taste with a cooling effect. It is traditionally recognized for its health-promoting effects, including improving eyesight, dispelling heat, quenching thirst, aiding digestion, and relieving bloating [3]. Notably, it exhibits significant effects in preventing food spoilage and preserving food during summer.

Recent studies have reported diverse pharmacological activities of hawk tea, such as hypoglycemic and hypolipidemic effects [4]. Combining the dual functions of “tea and medicine”, it offers substantial health benefits and high economic value. Furthermore, hawk tea is favored by consumers for its non-toxic, caffeine-free, side-effect-free, and pollution-free characteristics, highlighting its potential in functional food and natural healthcare applications.

The traditional processing technique of hawk tea is similar to that of green tea. The manufacturing process begins with high-temperature blanching of hawk tea leaves in an iron wok, followed by low-heat drying. During the drying stage, simultaneous rubbing and turning of the leaves are performed to yield the final hawk tea product. Due to the presence of volatile oils and polyphenolic compounds in hawk tea, it exhibits a distinct aroma characteristic of *Lauraceae* plants when brewed. Its strong flavor, which can be initially perceived as “unpleasant” by novice drinkers [2], poses a challenge to its broader acceptance. Additionally, hawk tea products tend to be rough in texture, lacking in aesthetic shaping, and of varying quality, resulting in poor appearance and inconsistent standards. This means that hawk tea has failed to meet market demands, which has significantly impacted its commercial value.

To address the issues of cheap product image and unpleasant taste, fermentation is employed to improve the flavor of food [5,6]. For example, *Eurotium cristatum* causes the conversion of phenolics initially present in unfermented green tea, adding desirable taste, color, and flavor features to the fermented tea product [7,8]. *Aspergillus cristatus*, the predominant microbe in Fuzhuan brick tea, is responsible for the creation of its distinctive golden flowers and unique floral aroma [9,10]. Therefore, the microbial fermentation process enables modification of flavor characteristics in tea leaves. Hawk tea contains substantial volatile components, like conventional tea leaves. It is anticipated that fermentation could also modify the composition and content of volatile compounds in hawk tea, thereby altering its flavor profile.

However, few studies have systematically investigated the use of specific microbial strains to modulate the flavor profile of hawk tea. To date, no microbial strain has been reported to reliably improve its sensory quality. Accordingly, this study aimed to select an optimal edible microorganism for the solid-state fermentation of hawk tea. We hypothesized that the unique enzymatic profiles and metabolic pathways of the selected microbe could transform hawk tea’s characteristic camphoraceous aroma into more desirable notes.

Four microbial strains commonly employed in food fermentation were selected based on their documented efficacy in flavor modulation and safety profiles, including *Monascus purpureus*, *Aspergillus cristatus*, *Bacillus subtilis*, and *Blastobotrys adeninivorans*. *Monascus purpureus* is known to enrich fermented products with ester and ketone compounds, creating distinct aromatic profiles [11]. *Aspergillus cristatus* has been shown to significantly alter the volatile profiles of dark teas and is considered a key contributor to the aroma of Fu brick tea [12,13]. *Bacillus subtilis*, a widely used safe strain, enhances flavor complexity in fermented beverages such as Baijiu [14]. *Blastobotrys adeninivorans*, essential in Pu-erh tea fermentation, exhibits broad substrate versatility and industrial potential [15].

Therefore, we used raw hawk tea materials as the fermentation substrate and four edible fungi as the fermentation strains. In the present study, we investigated the changes in volatile compounds during the solid-state fermentation process and identified the most suitable fungal strain for fermenting hawk tea through sensory evaluation. The findings of this study will lay a solid research foundation for the launch of new hawk tea products with enhanced flavor profiles.

## 2. Materials and Methods

### 2.1. Sample Preparation

The raw hawk tea materials were harvested in Wulong (Chongqing, China) and identified by Professor Zhang Dan (Chongqing University of Chinese medicine). The collected hawk tea was cut into small pieces and dried under 50 °C. *Monascus purpureus*, *Aspergillus cristatus*, *Bacillus subtilis*, and *Blastobotrys adeninivorans* were purchased from China Center of Industrial Culture Collection (CICC), which had preservation numbers CICC 40942, CICC 41701, CICC 21383, and CICC 33222, respectively. Potato dextrose agar (PDA) medium, yeast extract peptone dextrose (YPD), and nutrient broth (NB) medium was purchased from Hope Biol-Technology Co., Ltd. (Qingdao, China). Freeze-dried spores of *Monascus purpureus* CICC 40942, *Aspergillus cristatus* CICC 41701, and *Blastobotrys adeninivorans* CICC 33222 were recovered on PDA for 5 days at 32 °C and then cultured in YPD fluid medium at 180 rpm/min and 32 °C for 3 days. *Bacillus subtilis* CICC 21383 was recovered on PDA for 24 h at 35 °C and cultured in NB medium at 180 rpm/min at 35 °C for 2 days. After that, the concentration of seed broth was adjusted to 10^6^ spores/mL.

A mass of 30 g hawk tea was mixed with 30 mL distilled water in a 500 mL flask. The substrate was sterilized by autoclaving at 121 °C for 20 min, cooled to room temperature, and then inoculated with 10% (*v*/*w*) seed broth. The flasks were incubated statically at 32 °C (35 °C, *Bacillus subtilis*) for 12 days. After fermentation, the fermented products were dried by freeze-drying and powdered (100 mesh).

### 2.2. Electronic Nose Analysis

The volatile aroma profile was analyzed with a Heracles NEO ultra-fast GC e-nose (Alpha M.O.S., Toulouse, France). Exactly 0.5 g of each powdered sample was placed in a 20 mL headspace vial with 5 mL boiling water and equilibrated at 80 °C for 20 min. VOCs were then separated with two kinds of polar columns: a weak polar MXT-5 column and a medium polar MXT-1701 column (10 m × 180 µm × 0.4 µm, Restek, Bellefonte, PA, USA). And the temperature programs were conducted as follows: 40 °C to 130 °C at 1 °C/s, then to 180 °C at 1.5 °C/s, and finally to 210 °C at 0.3 °C/s. The trap temperature was 20 °C initially, and 240 °C at the end. The collection time of the trap was 40 s. The parameters for sample injection and detection were set as follows: injection volume, 5000 µL; injection rate, 250 µL/s; injection duration, 35 s; injector temperature, 200 °C; detector temperature, 260 °C; carrier gas, H_2_; FID gain, 12 [16]. Each batch was analyzed in triplicate. Compounds were identified by matching their retention index (RI), which was calibrated against the n-alkane standard (C6–C16) through the AroChemBase database.

### 2.3. Analysis of Hawk Tea Samples by GC-MS

GC-MS is widely recognized as a powerful analytical technique for both qualitative and quantitative analysis of VOCs, owing to its high sensitivity and capability to detect high-molecular-weight substances [10].

In this study, the analytical method was adapted from a reference with minor modifications [17]. Briefly, GC-MS analysis was performed using an Agilent 7890B-5977B GC-MS system (Agilent Technologies, Santa Clara, CA, USA). Chromatographic separation was achieved using an HP-5MS Ultra Inert capillary column (30 m × 250 μm × 0.25 μm) coupled with a PAL RSI 85 completely automated sample handling system to ensure consistent and efficient sample injection. For sample preparation, 0.5 g of tea sample, 0.5 g of sodium chloride, and 1 μL of cyclohexanone (0.236 mg/L, internal standard, Aladdin, Shanghai, China) were placed into a 20 mL headspace vial. Subsequently, 5 mL of boiling ultrapure water was added, and the vial was equilibrated for 5 min before being tightly sealed. The vial was then incubated at 60 °C for 1 h to facilitate the release of volatile compounds. Then, volatiles were extracted for 1 h at 60 °C with a 50 µm DVB/CAR/PDMS SPME fiber (Supelco, Sigma-Aldrich, Bellefonte, PA, USA). After extraction, the fiber needle was immediately inserted into the GC injector and desorbed at 250 °C for 1 min. High-purity helium served as the carrier gas at a constant flow rate of 1 mL/min with no ratio. The column temperature was initially held at 40 °C for 5 min, ramped up to 100 °C at 4 °C/min, then to 180 °C at 2 °C/min, and finally to 250 °C at 10 °C/min and held for 5 min. MS was in electron impact (EI) mode at 70 eV; ion source and quadrupole temperatures were 230 °C and 150 °C, respectively. The solvent delay was set to 3 min, and the mass scanning range was 35–550 *m*/*z*.

The RI of each volatile compound was calculated using the retention times of a C7–C30 n-alkanes standard mixture. Compound identification was performed by comparing the mass spectra with those in the NIST 17.0 database, and only compounds with a similarity index greater than 80% were considered valid.

### 2.4. Analysis of Hawk Tea Samples by GC-IMS

The VOCs in samples were also analyzed using a GC-IMS system (FlavourSpec^®^, G.A.S., Dortmund, Germany). The analytical method was adapted from a reference with minor modifications [18]. In brief, 0.5 g of each sample with 5 mL boiling water was added into a 20 mL headspace vial and incubated at 500 rpm for 20 min at 60 °C, with 10 μL 2-methyl-3-heptanone (100 mg/L) as internal standard. After incubation, 500 μL of the headspace gas was automatically injected into the GC-IMS system using a heated syringe needle maintained at 85 °C. Separation was performed on the MXT-WAX capillary column (30 m × 0.53 mm × 1 μm, Restek, USA). The carrier gas was high-purity nitrogen (purity ≥ 99.99%) and flow rate protocol was as follows: the speed started at 2 mL/min for 2 min, and was ramped up to 10 mL/min in 8 min, which was then followed by an increase to 100 mL/min in 10 min, which then held for 40 min. The drift tube length was 53 mm, and the stable drift gas flow rate was 75 mL/min. The temperature of the drift tube was set to 45 °C. Similarly, the RI of VOCs was determined by using n-ketones (C4–C9) as a standard solution, while qualitative analysis was conducted on the VOCs in samples via the built-in NIST 2020 and IMS databases. Each batch of samples was measured three times in parallel.

### 2.5. Calculation of Odor Activity Value and Relative Odor Activity Value

To evaluate the influence of various volatile compounds on the overall aroma profile of hawk tea, the OAVs and ROAVs were calculated. In the literature, OAV is defined as the ratio of the concentration of a specific volatile compound to its corresponding odor detection threshold. An OAV greater than 1 indicates that the compound plays a significant role in contributing to the overall aroma of the sample [19]. Meanwhile, the ROAV of the volatile flavor compound that most obviously affected the overall flavor of the sample was set to 100. Compounds with ROAV ≥ 1 were classified as key aroma-active volatiles, whereas those with 0.1 < ROAV < 1 were regarded as aroma modifiers [20].

The formula for calculating OAV is

OAVi = Ci/Ti

OAVi is the odor activity of the compound, Ci represents the concentration of the compound, and Ti is the threshold of the compound in water.

The formula for the relative odor activity value (ROAV) [19] is as follows:

ROAVi = (Ci × Tmax) × 100/(Ti × Cmax)

ROAVi is the relative odor activity value of the compound, Ci represents the relative concentration of the compound, Ti represents the threshold of the compound in water, and Cmax/Tmax represents the highest Ci/Ti ratio observed among all constituents of the sample.

In OAV and ROAV calculations, the mean concentration or relative content of a compound serves as the input parameter.

### 2.6. Sensory Evaluation

Based on preliminary experiments and the consensus on ideal tea flavor, we established a clear sensory optimization objective. The goal was to develop a product with more appealing color, enhanced desirable aroma (such as fresh, floral, sweet notes), and more complex overall aroma, while avoiding or reducing off-flavors such as bitterness and astringency. Sensory evaluation analysis of hawk teas followed the method of Rodríguez-Noriega et al. [21] with minor modifications. Sample preparation: the names of tea samples were labeled with three-digit random codes and presented to each panelist in a randomized order. Each sample was equally distributed on trays for visual assessment of appearance characteristics, including color, integrity, and clarity of the leaves. After that, 3 g dry tea leaves was added to 150 mL of boiled water for 5 min. The infused tea was evaluated for aroma, color, and taste. Panelist evaluation procedure: the sensory evaluation panel consisted of nine individuals (eight females and one male) with no known sensory impairments and no prior formal training in sensory analysis. Before the evaluation, panelists received a brief introduction to the Flash Profile method, covering the main goals and general procedure. A set of reference terms was also provided to support them in developing sensory descriptors. During the first session, all tea samples were presented simultaneously in a randomized order. Panelists were asked to freely give sensory descriptors related to the samples’ appearance, liquor color, aroma, taste, and infused leaf, with no restriction on the number of attributes. The descriptors generated by each panelist were collected and merged into a shared list of sensory terms. In the following session, panelists reviewed this list, refined their personal descriptor sets, and then used them to evaluate and rank the samples based on the perceived intensity of each attribute.

### 2.7. Statistical Analysis

R (v 4.3.2), Metware cloud platform (https://cloud.metware.cn; accessed on 25 September 2025), Origin 2021, and XLSTAT 2019 were used for statistical analysis and data visualization. The statistical analysis was performed using one-way analysis of variance (ANOVA), and Duncan’s multi-range test was run with SPSS Statistics 22 (IBM, Armonk, NY, USA). Values of *p* < 0.05 and VIP > 1 were regarded as statistically significant. The GC-IMS results were qualitatively analyzed using VOCal (v 0.4.03) and its plug-ins.

## 3. Results and Discussion

### 3.1. Flavor Profiles of Hawk Tea Samples Revealed by Electronic Nose

In this study, an ultra-fast GC e-nose was employed to analyze the volatile profile of hawk tea fermented by different microbial strains. Unlike conventional e-noses that rely on sensor arrays, this system separates odorants chromatographically, enabling rapid and sensitive detection of individual compounds [22]. Volatile separation was achieved using two capillary columns with distinct polarities (MXT-5 and MXT-1701), as shown in Figure S1. By matching RI with the ArochemBase database, a total of 31 aroma-active compounds were identified, comprising 7 alcohols, 3 aldehydes, 3 ketones, 7 esters, 7 terpenes, and 4 other compounds (Table S1 for details).

**Figure 1:**
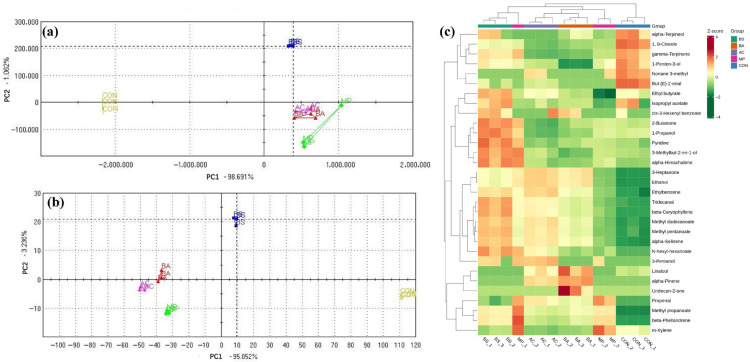
The volatile compounds in hawk tea samples with different fermentation strains based on electronic nose data. (**a**) PCA; (**b**) DFA; (**c**) heat map of VOCs. AC group: hawk tea fermented with *Aspergillus cristatus*; BA group: hawk tea fermented with *Blastobotrys adeninivorans*; BS group: hawk tea fermented with *Bacillus subtilis*; MP group: hawk tea fermented with *Monascus purpureus*; CON group: hawk tea with no fermentation.

To explore the aroma-based distinction among hawk tea samples, principal component analysis (PCA) followed by discriminant function analysis (DFA) was conducted. PCA was used to reduce data dimensionality while preserving the most informative variance patterns [23]. DFA, on the other hand, was applied to enhance class separability by maximizing inter-group variance and minimizing intra-group scatter [24]. The first two principal components captured 99.84% of the total variance in PCA (Figure 1a), and 98.28% in DFA (Figure 1b), meaning that they were responsible for most of the key fragrance features of the various hawk tea samples. Both models successfully distinguished hawk tea samples according to their aroma profiles. In Figure 1c, the relative contents of 1-penten-3-ol and 1,8-cineole decreased markedly in fermented hawk tea. This shift has been reported to potentially reduce undesirable oxidative and camphoraceous flavors in hawk tea [25,26].

### 3.2. The Volatile Compounds of Hawk Tea Samples Identified by GC-MS

GC-MS was used to identify and quantify volatile compounds in hawk tea and samples fermented with different microbial strains. A total of 74 volatile compounds were detected across the five hawk tea samples, including 1 aldehyde, 2 ketones, 17 alcohols, 3 esters, 48 alkenes and 3 other compounds. The total ion chromatograms (TICs) and detailed information on the volatile compounds for each sample are shown in Figure S2 and Table 1.

**Figure 2:**
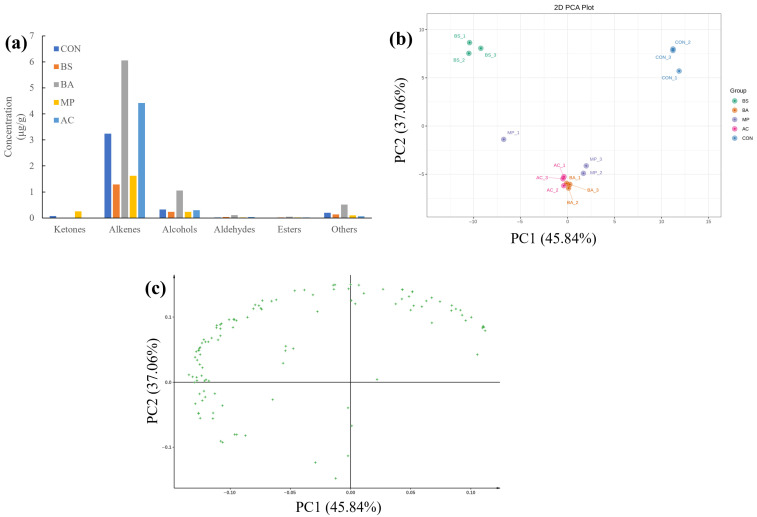
The volatile compounds in hawk tea samples with different fermentation strains based on GC-MS data. (**a**) Changes in volatile substances; (**b**) PCA plot; (**c**) PCA loading. AC group: hawk tea fermented with *Aspergillus cristatus*; BA group: hawk tea fermented with *Blastobotrys adeninivorans*; BS group: hawk tea fermented with *Bacillus subtilis*; MP group: hawk tea fermented with *Monascus purpureus*; CON group: hawk tea with no fermentation.

Table 1 shows that the contents and types of volatile compounds changed in hawk tea after fermentation. Specifically, alcohols and alkenes in the samples displayed significant variations. For instance, samples in the BS group (*Bacillus subtilis*-fermented tea) and MP group (*Monascus purpureus*-fermented tea) had lower contents of and fewer alcohols and alkenes compared to the unfermented raw hawk tea materials (CON group). In contrast, alcohols and alkenes in the *Blastobotrys adeninivorans*-fermented tea (BA group) increased to 3.26 times and 1.87 times the levels in the CON group, respectively (Figure 2a).

Alkenes were the most abundant in both quantity and variety among the hawk tea samples, with terpenes being the predominant type. Terpenes are a widely occurring class of natural products with important physiological functions and significant bioactivities, having broad applications in food, medicine, and cosmetics. Their main biosynthetic pathways are the methylerythritol phosphate (MEP) and mevalonate (MVA) pathways [27]. Notably, gamma-muurolene was not detected in the BA group but was present in all other samples. Conversely, gamma-gurjunene was found in the BA group at 0.43 ± 0.422 μg/g but was not detected in other samples. It is speculated that *Blastobotrys adeninivorans* may effectively alter the carbon skeleton of cyclic terpenes [28], and might increase the musty odor in hawk tea. Other terpenes showing significant changes include gamma-cadinene and humulene. Gamma-cadinene increased from 0.145 ± 0.03 μg/g in the CON group to 0.571 ± 0.468 μg/g in the BA group, which may enhance the herbal and woody odor of hawk tea. Humulene was not detected in the CON group but was found in all fermented samples. This likely contributes to the stronger woody flavor in fermented hawk tea [29].

Alcohols accounted for the second largest proportion of volatile compounds in hawk tea. They are important sources of characteristic aromas in tea [30,31]. In this study, the total alcohol content decreased in the BS, AC, and MP groups compared to the CON group. However, the total alcohol content in the BA group exhibited a three-fold increase compared with that in the CON group. Alcohols like cubebol, zingiberenol, and especially eudesmol isomers increased in the fermented samples. For example, gamma-eudesmol increased from 0.051 ± 0.028 μg/g in the CON group to 0.118 ± 0.011 μg/g in the BA group, which may enhance woody and sweet aromatic notes [32,33]. Cubebol increased from 0.033 ± 0.022 μg/g in the CON group to 0.103 ± 0.007 μg/g in the BA group, potentially adding to the cooling sensation of the BA-fermented tea [34].

Three ester compounds were identified in the GC-MS analysis. Notably, the bornyl acetate content increased in all fermented groups. Bornyl acetate has a cooling herbal, pine needle-like aroma, evoking a fresh forest scent [35]; it suggests that fermentation may enhance green-refreshing notes in hawk tea.

The PCA analysis of the volatile components (Figure 2b,c) shows that hawk tea samples fermented with the four different microbes could be clearly distinguished, indicating that they developed distinct flavor characteristics.

### 3.3. The Volatile Compounds of Hawk Tea Samples Identified by GC-IMS

Hawk tea and its fermented products were also analyzed using GC-IMS, owing to several advantages: the short analysis time, simple sample preparation, and visualization of the results [36].

**Figure 3:**
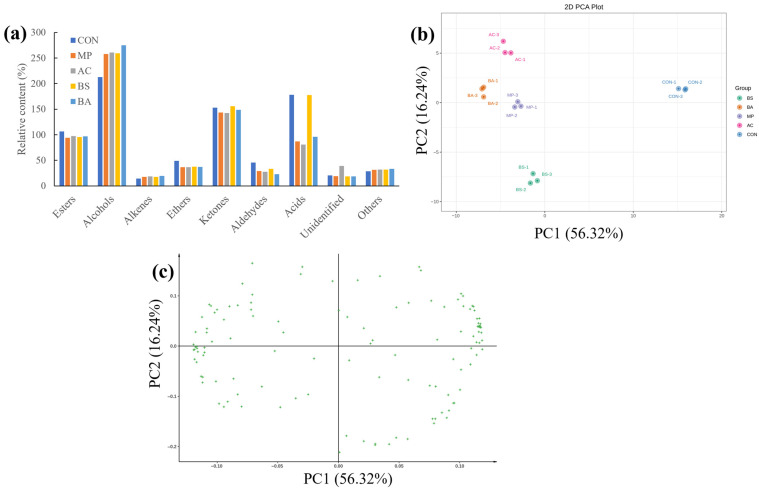
The volatile compounds in hawk tea samples with different fermentation strains based on GC-IMS data. (**a**) Changes in volatile substances; (**b**) PCA plot; (**c**) PCA loading. AC group: hawk tea fermented with *Aspergillus cristatus*; BA group: hawk tea fermented with *Blastobotrys adeninivorans*; BS group: hawk tea fermented with *Bacillus subtilis*; MP group: hawk tea fermented with *Monascus purpureus*; CON group: hawk tea with no fermentation.

Although some compounds identified by GC-MS and GC-IMS were the same, most of the detected compounds were different due to the different sensitivities of the two methods. GC-IMS is more suitable for detecting smaller volatile molecules. Combining the two detection methods provides a more complete picture of the volatile compounds in hawk tea samples. In this study, 121 peak signals were detected, and, in total, 103 compounds were identified (Table S2). These included monomers and dimers of volatile substances (represented by the letters M for monomer and D for dimer). The 103 compounds consisted of 4 acids, 19 esters, 26 alcohols, 21 aldehydes, 14 ketones, 13 alkenes, 3 ethers, and 3 other types. Meanwhile, the fermented hawk tea generally exhibited a reduction in compounds such as esters, ethers, aldehydes, and acids (except in the BS group), alongside an increase in terpenes and alcohols (Figure 3a). These two kinds of aromatic compounds have been reported to provide floral and fruity attributes, which have a good coordinating effect on the aroma profiles of tea products [37,38].

Then, the contents of the 103 VOCs were subjected to PCA to discriminate the samples (Figure 3b,c). The figures show that the five types of hawk tea could be clearly separated. The first two principal components (PC1 and PC2) for hawk tea explained 72.56% of the total variation, indicating that these two components captured most of the sample information. This result agrees with the earlier discussion based on GC-MS data.

**Figure 4:**
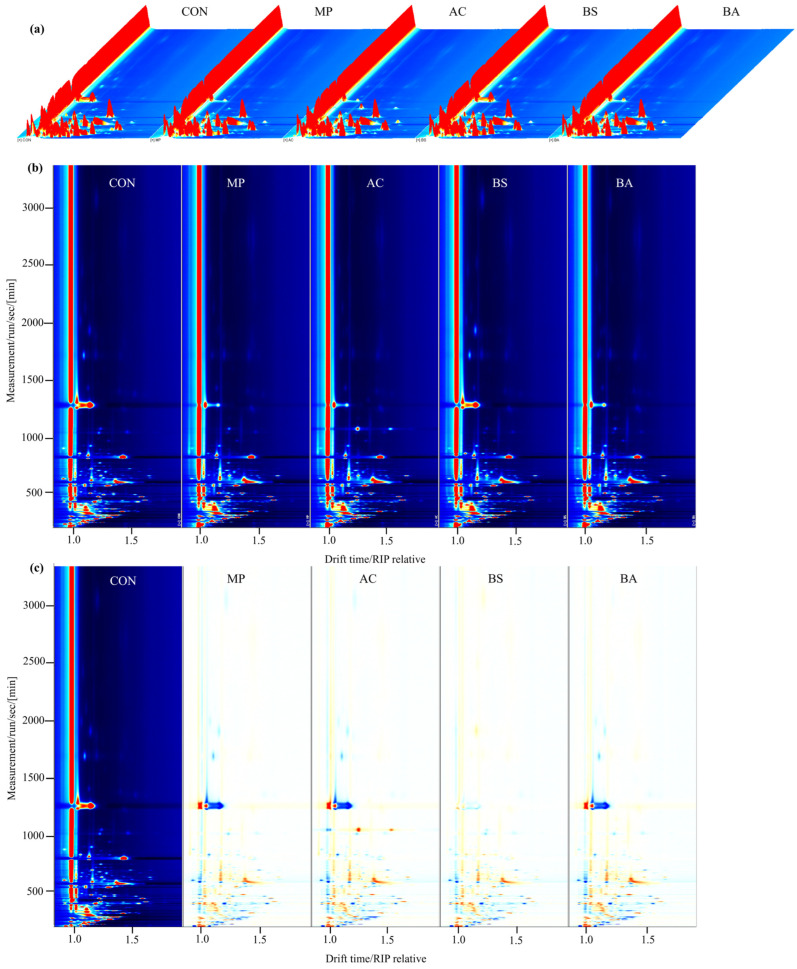
The volatile compounds in hawk tea samples with different fermentation strains based on GC-IMS data. (**a**) 3D topographic plot. (**b**) Airscape maps. (**c**) Contrast maps. AC group: hawk tea fermented with *Aspergillus cristatus*; BA group: hawk tea fermented with *Blastobotrys adeninivorans*; BS group: hawk tea fermented with *Bacillus subtilis*; MP group: hawk tea fermented with *Monascus purpureus*; CON group: hawk tea with no fermentation.

Subsequently, three-dimensional (3D) plots were used to show the differences and changes in VOCs among different hawk tea samples. As illustrated in Figure 4a, the peak signal distributions of volatile compounds exhibited similarity across hawk tea samples fermented with distinct microbial strains. However, the peak signal intensities differed among the samples. This demonstrates that while similar VOC profiles were present across hawk tea samples, their relative abundance exhibited microbial-specific variations. To enable visual comparison of volatile compound variations, the two-dimensional (2D) spectrum of the CON group served as the reference. Differential spectra were generated by subtracting other sample spectra from this reference, producing comparative contour plots (Figure 4b,c). In these differential plots, a white background signifies an identical VOC level between the sample and reference. Regions in red represent compounds with elevated concentrations in the sample, whereas blue regions indicate decreased levels. Consequently, these differential plots clearly demonstrate distinct alterations in volatile compound concentrations within hawk tea following microbial fermentation.

**Figure 5:**
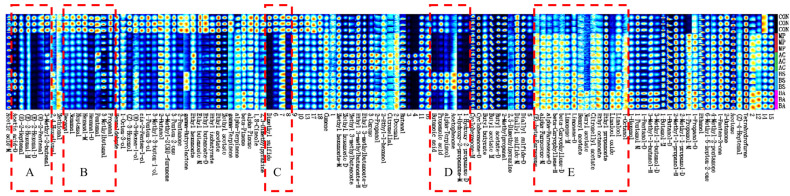
Volatile compound fingerprints of hawk tea samples fermented with different strains based on GC-IMS data. AC group: hawk tea fermented with *Aspergillus cristatus*; BA group: hawk tea fermented with *Blastobotrys adeninivorans*; BS group: hawk tea fermented with *Bacillus subtilis*; MP group: hawk tea fermented with *Monascus purpureus*; CON group: hawk tea with no fermentation. The red dashed frames highlight these differential regions for visual clarity. A–E: characteristic volatile regions that were significantly influenced by fermentation.

To complement the spectral data and enable a detailed comparison of VOCs across the five hawk tea samples, ion mobility fingerprint spectra were generated (Figure 5). This approach provides specific compound information that cannot be accurately derived from the 3D and 2D spectra alone. The horizontal axis represents the different compounds detected in the samples, and the vertical axis represents the three replicates for each sample. The brightness corresponds to the compound response value, with greater brightness indicating a higher content. By organizing and summarizing the characteristic compounds in hawk tea samples subjected to different strains through fingerprint spectra, preliminary clustering can be achieved.

In boxes A, B and C (Figure 5), the relative levels of several VOCs associated with fatty, pungent, and sour notes decreased in the fermented groups compared to the control. These included (E)-2-pentenal (pungent, green), (E)-2-hexenal-D (green, banana, fat), 3-methyl-2-butenal (sweet, fruity, yet pungent and nutty), hexanal-D and acetic acid-D (sharp, sour, except the BS group), dimethyl sulfide (sulfur, gasoline). Conversely, shown in box D, fermentation with *Bacillus subtilis* (BS group) led to a marked increase in compounds contributing to harsh and cheesy aromas, such as 1-hydroxy-2-propanone (pungent, sweet), acetophenone (sweet, almond-like), butanoic acid (cheesy, rancid), and propanoic acid (pungent, acidic). These alterations in volatile composition suggest that microbial fermentation generally moderated the harsh off-odors in raw hawk tea. As shown in box E, fermentation significantly elevated the levels of alkenes, alcohols, and esters, in the other three groups. It included a substantial increase in compounds contributing to fresh and fruity aromas, such as limonene (citrus), beta-caryophyllene (woody), ethyl octanoate (fruity), and citronellyl acetate (floral). The enrichment of these odorants imparts a more fruity and fragrant character to the aroma profile of the fermented hawk tea. However, *Bacillus subtilis*-fermented hawk tea displayed a distinct and dominant acidic character, which is typically considered an undesirable attribute for tea aroma. Therefore, based on the aroma profile derived from the volatile compound data, *Bacillus subtilis* may not be the most suitable microbial strain for hawk tea fermentation when the primary goal is aroma improvement.

### 3.4. Odor Profiles of Five Hawk Tea Samples

The contribution of an individual volatile to the overall aroma of hawk tea depends on its concentration and odor threshold, and the contribution is determined by its odor activity value (OAV) [18]. According to the concentrations and thresholds of VOCs (Table 1), six volatile compounds exhibited OAVs greater than 1 among five hawk tea samples (Table 2). Similar to the OAV, the ROAV often serves for the assessment of the effect on the overall aroma of individual volatile compounds [39]. Since the OAV requires the absolute concentrations which are not always available, the ROAV has become a common alternative for comparing aroma contribution across samples. According to the data in Table S2, 25 volatile compounds exhibited ROAVs greater than 1 in five hawk tea samples (Table 3).

(Z)-beta-ocimene is one of the natural isomers of ocimene (3,7-dimethyl-1,3,6-octatriene), which is a volatile mono terpene with a grassy and floral scent [40]. In this study, (Z)-beta-ocimene was detected only in the CON group. This indicated that fermentation progress decreased the concentration of ocimene, and this reduction may diminish the grassy odor of fermented hawk teas. Beta-caryophyllene is a plant terpenoid with therapeutic and biofuel properties [41], exhibiting sweet, woody, spice, clove, and dry notes [42]. In BA and AC groups, OAVs of beta-caryophyllene displayed a slight increase compared with the CON group. Furthermore, the comparison of the bornyl acetate OAVs across all samples revealed that the woody note attribute contributed by bornyl acetate [43] was substantially more prominent in the overall aroma profile of fermented samples. This indicates that fermented hawk teas may develop a more pronounced woody note and a richer, mellower profile. Of particular significance is the marked high OAVs in perillaldehyde and ethyl decanoate in the BA group, contributing distinct floral–fruity notes [44,45].

Then, as shown in Table 3, fermented hawk teas exhibited a consistent shift toward a more desirable aroma profile. Compounds associated with pleasant notes, such as 3-methyl-1-butanol (malt, whiskey, banana), 1-hexanol (fresh, fruity), and linalool (citrus, floral) [46,47,48], showed much higher ROAVs than the CON group. And this means that they have a greater contribution to the aroma, particularly in the BA group. In contrast, the fatty and camphoraceous notes characteristic of the raw tea were attenuated after fermentation. This was reflected in the decreased ROAVs of 1,8-cineole (eucalyptus, camphor) and nonanal (oil oxidation, citrus) [47,49].

Based on the OAV and ROAV data, the BA group demonstrated a more complex and intense aromatic profile, forming a fuller and more layered flavor. Overall, fermented tea exhibits a stronger woody, floral, and fruity aroma compared to unfermented hawk tea.

### 3.5. Sensory Evaluation of Hawk Tea Flavor

The sensory panelists generated more than forty descriptors encompassing appearance, liquor color, aroma, taste, and brewed leaves. In the Flash Profile method, where descriptors were elicited individually by each assessor, this process resulted in some overlapping or ambiguous terms (such as “minty” and “Chinese herbal medicine”). Following panel discussions, correlated descriptors were consolidated, resulting in a final set of 16 candidate sensory descriptors for hawk tea (Table 4). Subsequently, panelists autonomously selected descriptors from this pool that they could perceptibly discriminate, developed individualized sensory lexicons, and proceeded to evaluate the hawk tea samples.

**Figure 6:**
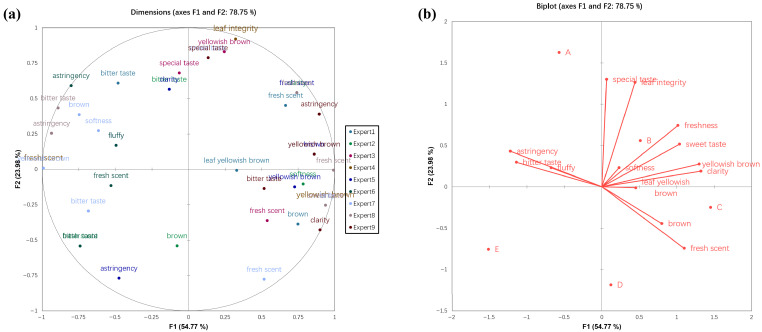
Sensory analysis of hawk tea samples with different fermentation strains. (**a**) Sample factor loading plots of GPA. (**b**) Sensory attribute loading plots of GPA. A represents BS group, B represents BA group, C represents MP group, D represents AC group, E represents CON group.

Following generalized Procrustes analysis (GPA) of the Flash Profile data, sample factor loading plots and sensory attribute loading plots were generated (Figure 6). The first two principal dimensions (F1 and F2) cumulatively accounted for 78.75% of the sensory data variation, providing a reliable basis for assessing sensory differences among samples. The consensus index (Rc) for the panelists was 51.2%. The Rc value was close to the value reported by Rodríguez-Noriega et al. [21], and the value indicated sufficient consensus among the panelists on the hawk tea samples.

Within the consensus space (Figure 6b), the samples exhibited distinct distribution patterns, revealing significant sensory differences among the treatment groups. Sample B (BA group) was positioned in the upper-right quadrant of the spatial map, indicating that its sensory profile was most closely associated with the vectors for positive attributes such as sweetness and fresh scent. In contrast, Sample A (BS group) was located on the left side of the space, with its position linked to the attributes of bitterness and astringency. Sample C (MP group) and Sample D (AC group) were clustered in the lower-left region, both characterized by dark coloration and distanced from vectors representing aromatic complexity. Sample E (CON, control group) was situated near the center of the plot and was not strongly associated with any dominant attributes, thereby establishing the sensory evaluation baseline.

Combined with the pre-defined flavor optimization objective, the spatial position of Sample B (BA group) clearly demonstrates its closest alignment with the goal of “enhancing pleasant color, desirable aroma, and complex overall aroma.” Consequently, from the perspective of descriptive sensory analysis, *Blastobotrys adeninivorans* was identified as the most promising strain for improving the flavor profile of hawk tea. This sensory assessment was consistent with the results from volatile compound analysis using GC-MS and GC-IMS. The BA group was found to contain a richer profile and higher concentrations of key pleasant aroma compounds (perillaldehyde, ethyl decanoate, linalool), whereas the BS group was likely associated with a higher presence of compounds related to undesirable flavors.

The hierarchical cluster analysis (HCA) results for the five hawk tea samples are presented in Figure S3. The HCA clearly segregated the samples into two distinct clusters: non-fermented and fermented. This pronounced separation indicates significant modifications in the sensory characteristics induced by microbial fermentation. Furthermore, the sensory panelists employing the Flash Profile methodology demonstrated the capacity to rapidly and accurately differentiate between the fermented and non-fermented hawk tea products based on these distinctive sensory profiles.

### 3.6. The Key Aroma Compounds in Blastobotrys adeninivorans-Fermented Hawk Tea

**Figure 7:**
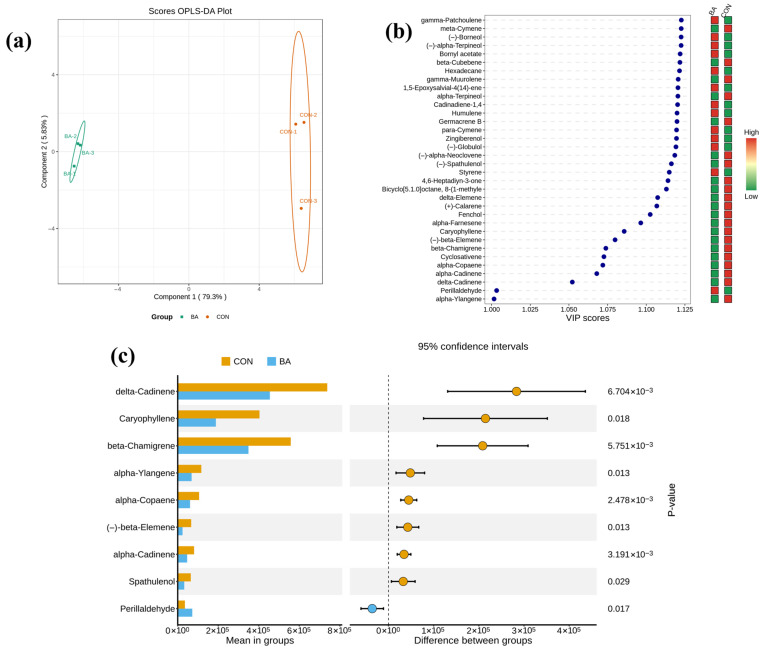
Comparison between BA group and CON group with GC-MS data. (**a**) OPLS-DA plot; (**b**) VIP scores and hot map; (**c**) *p* values. BA group: hawk tea fermented with *Blastobotrys adeninivorans*; CON group: hawk tea with no fermentation.

Based on the above analyses, *Blastobotrys adeninivorans* is more suitable as a fermentative strain for improving the flavor of hawk tea. A further orthogonal partial least squares discriminant analysis (OPLS-DA) of the GC-MS and GC-IMS datasets revealed differential aroma compounds between the BA and CON groups. OPLS-DA was typically applied for discriminant analysis and the screening of differential compounds, which proved to be a powerful tool [16]. By integrating VIP values and p-values, the characteristic aroma constituents of the BA group were identified. As shown in Figure 7, the samples in the CON and BA groups are clearly separated. For GC-MS, variables with VIP > 1 and *p* < 0.05 were deemed to discriminate significantly between the two groups; among them, compounds whose OAVs exceeded 1 were taken as aroma markers. Consequently, perillaldehyde was identified as a characteristic aroma compound of *Blastobotrys adeninivorans*-fermented hawk tea, which contributes distinct floral and fruity notes to the sensory profile.

**Figure 8:**
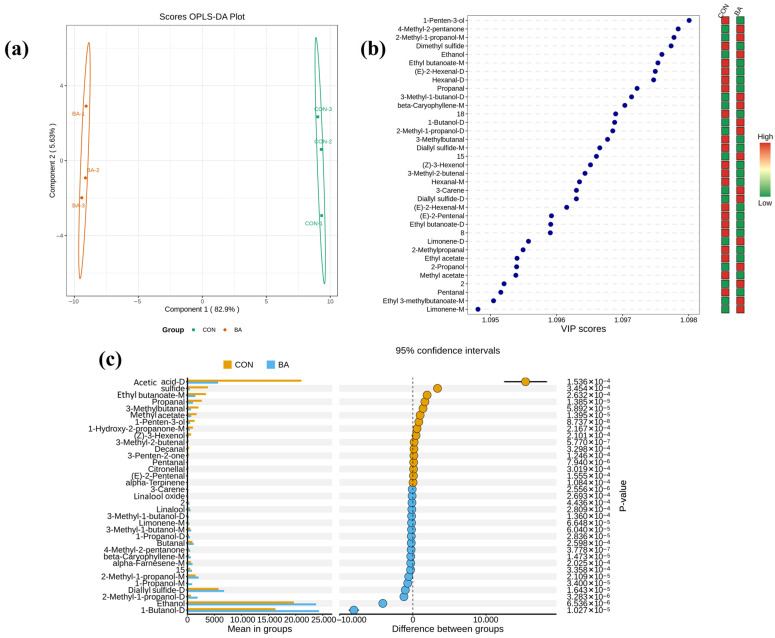
Comparison between BA group and CON group with GC-IMS data. (**a**) OPLS-DA plot; (**b**) VIP scores and hot map; (**c**) *p* values. BA group: hawk tea fermented with *Blastobotrys adeninivorans*; CON group: hawk tea with no fermentation.

For GC-IMS, the top 35 volatile compounds were visualized in Figure 8 (VIP > 1 and *p* < 0.05). Compounds with ROAV > 1 and an increasing trend after *Blastobotrys adeninivorans* fermentation were regarded as key aroma-active compounds; then, nine such compounds were selected, namely, butanal (pungent, fruity, green leaf), butyl acetate-M (fruity), diallyl sulfide-D (garlic), 1-butanol-D (wine), 3-methyl-1-butanol-D (whiskey, banana, fruity), 3-methyl-1-butanol-M (whiskey, banana, fruity), 1-hexanol (fresh, fruity, wine, sweet, green), linalool (citrus, rose, woody, blueberry), and 1-penten-3-one (strong pungent odors). The ROAV of linalool was 100, the largest among these nine compounds. This indicates that *Blastobotrys adeninivorans* fermentation enhances the fruity and woody aroma notes of hawk tea.

Therefore, fermentation with *Blastobotrys adeninivorans* significantly enhanced the fruity and floral character of hawk tea. The sensory attributes are directly linked to the key chemical markers perillaldehyde (OAV 3.692, floral, fruity) and linalool (ROAV 100, citrus, rose, woody, blueberry), which collectively build the complex aromatic profile of the fermented product. These results strongly support the findings of the sensory evaluation.

## 4. Conclusions

The primary aim of this study was to screen the most suitable microbial strain among four different microorganisms for improving the flavor quality of hawk tea through fermentation. By integrating artificial sensory evaluation with electronic nose, GC-MS and GC-IMS analysis, *Blastobotrys adeninivorans* was identified as the optimal fermentation strain for hawk tea. *Blastobotrys adeninivorans* significantly enhanced the floral and fruity aroma and mellow taste of hawk tea while effectively reducing its characteristic camphor-like odor. These findings provide a solid foundation for flavor optimization of hawk tea and offer promising possibilities for the development of new hawk tea products. Additionally, the results highlight that different analytical methods target different ranges of volatile compounds, suggesting that a combination of multiple techniques is the most effective approach for comprehensive flavor analysis.

While this study successfully screened a promising microbial candidate for flavor enhancement, it primarily focused on establishing the proof of concept at the laboratory scale. Future research should aim to optimize the fermentation process parameters for potential scale-up, validate the sensory improvements through consumer acceptance studies, and employ advanced multi-omics methodologies to deepen the understanding of the underlying metabolic mechanisms. Such investigations would further bridge the gap between laboratory discovery and practical application, ultimately facilitating the development of high-quality, consumer-accepted fermented hawk tea products.

## Figures and Tables

**Table 1:** Volatile compounds identified in raw hawk tea materials and fermented teas processed by solid-state fermentation via GC-MS analysis (*n* = 3).

No.	Compounds	RI ^a^	RI ^b^	CAS	Odor ^c^	Thresholds (mg/kg) ^d^	Content (μg/g)				
							CON	BS	**BA**	**MP**	**AC**
	**Ketones**										
1	4,6-Heptadiyn-3-one	868	868	29743-27-9	-	n.f.	0.052 ± 0.017	0.012 ± 0.006	n.d.	0.254 ± 0.059	n.d.
2	1,3-Dichloroacetone	862	868	534-07-6	-	n.f.	0.026 ± 0.021	n.d.	n.d.	n.d.	n.d.
	**Alkenes**										
3	Sabinene	974	974	3387-41-5	Woody, terpene, citrus, pine, spice	0.98	0.009 ± 0.004	n.d.	n.d.	n.d.	n.d.
4	meta-Cymene	1023	1023	535-77-3	-	0.8	0.015 ± 0.001	n.d.	n.d.	n.d.	n.d.
5	Isosylvestrene	1027	1027	1461-27-4	-	n.f.	0.059 ± 0.018	n.d.	0.162 ± 0.014	n.d.	0.154 ± 0.008
6	(Z)-beta-Ocimene	1039	1038	3338-55-4	Warm, floral, herb, flower, sweet	0.034	0.05 ± 0.043	n.d.	n.d.	0.004 ± 0.001	n.d.
7	Bicyclo[5.1.0]octane, 8-(1-methyle	1098	1099	54166-47-1	-	n.f.	0.006 ± 0.001	n.d.	n.d.	n.d.	n.d.
8	delta-Elemene	1336	1338	20307-84-0	Sweet, herbal, woody	n.f.	0.022 ± 0.008	n.d.	n.d.	n.d.	0.006 ± 0.005
9	Cyclosativene	1365	1368	22469-52-9	-	n.f.	0.003 ± 0	n.d.	n.d.	n.d.	n.d.
10	alpha-Ylangene	1369	1372	14912-44-8	-	n.f.	0.073 ± 0.008	0.025 ± 0.002	0.105 ± 0.021	0.033 ± 0.011	0.081 ± 0.027
11	alpha-Copaene	1374	1376	3856-25-5	Woody, spicy, honey	n.f.	0.065 ± 0.004	0.029 ± 0.002	0.093 ± 0.013	0.034 ± 0.013	0.102 ± 0.008
12	(−)-beta-Elemene	1391	1391	515-13-9	Sweet	n.f.	0.041 ± 0.007	0.013 ± 0.002	0.036 ± 0.007	0.015 ± 0.005	0.037 ± 0.008
13	beta-Caryophyllene	1417	1419	87-44-5	Sweet, woody, spice, clove, dry	0.064	0.252 ± 0.038	0.104 ± 0.014	0.291 ± 0.031	0.119 ± 0.034	0.27 ± 0.054
14	Isosativene	1427	1428	24959-83-9	-	n.f.	n.d.	n.d.	0.01 ± 0.004	n.d.	n.d.
15	(+)-Calarene	1432	1432	17334-55-3	-	n.f.	0.008 ± 0.002	n.d.	n.d.	n.d.	0.009 ± 0.007
16	(−)-alpha-Neoclovene	1454	1454	4545-68-0	-	n.f.	0.004 ± 0	n.d.	n.d.	n.d.	n.d.
17	Alloaromadendrene	1463	1461	25246-27-9	Woody	n.f.	0.013 ± 0.006	n.d.	0.144 ± 0.101	n.d.	0.038 ± 0.01
18	gamma-Gurjunene	1474	1473	22567-17-5	Musty	n.f.	n.d.	n.d.	0.43 ± 0.422	n.d.	n.d.
19	gamma-Muurolene	1478	1477	30021-74-0	Herbal, woody, spice	n.f.	0.181 ± 0.039	0.085 ± 0.112	n.d.	0.102 ± 0.075	0.304 ± 0.231
20	beta-Selinene	1484	1486	17066-67-0	Herbal,	n.f.	0.513 ± 0.096	0.325 ± 0.09	0.862 ± 0.093	0.34 ± 0.081	0.648 ± 0.128
21	Guaiene	1490	1490	88-84-6	Sweet, woody, dry, guaiacwood, spicy, powdery	n.f.	0.076 ± 0.041	0.042 ± 0.027	0.106 ± 0.029	0.051 ± 0.024	0.12 ± 0.007
22	(−)-alpha-Muurolene	1499	1499	10208-80-7	Woody	n.f.	0.126 ± 0.022	0.059 ± 0.011	0.344 ± 0.262	0.062 ± 0.014	0.133 ± 0.01
23	gamma-Cadinene	1512	1513	39029-41-9	Herbal, woody	n.f.	0.145 ± 0.03	0.107 ± 0.027	0.571 ± 0.468	0.09 ± 0.029	0.215 ± 0.031
24	delta-Cadinene	1522	1524	483-76-1	Thyme, herbal, woody, dry	n.f.	0.461 ± 0.043	0.278 ± 0.037	0.703 ± 0.101	0.279 ± 0.084	0.633 ± 0.09
25	Zonarene	1532	1527	41929-05-9	-	n.f.	0.044 ± 0.027	n.d.	n.d.	n.d.	n.d.
26	alpha-Cadinene	1538	1538	24406-05-1	Woody, dry	n.f.	0.05 ± 0.004	0.031 ± 0.005	0.071 ± 0.01	0.035 ± 0.007	0.061 ± 0.009
27	alpha-Calacorene	1540	1542	21391-99-1	Woody	n.f.	0.034 ± 0.017	0.051 ± 0.012	0.149 ± 0.004	0.042 ± 0.013	0.063 ± 0.024
28	Germacrene B	1553	1557	15423-57-1	Woody, earthy, spicy	n.f.	0.011 ± 0.002	n.d.	n.d.	n.d.	n.d.
29	trans-Sesquisabinene hydrate	1584	1581	145512-84-1	-	n.f.	n.d.	n.d.	n.d.	0.012 ± 0.01	0.016 ± 0.007
30	Cadalene	1672	1674	483-78-3	-	n.f.	n.d.	0.009 ± 0.006	n.d.	n.d.	0.006 ± 0.001
31	para-Cymene	1024	1025	99-87-6	Fresh, citrus, terpene, woody, spice	0.005	n.d.	0.011 ± 0	0.027 ± 0.004	0.011 ± 0.001	0.02 ± 0.011
32	beta-Cubebene	1388	1389	13744-15-5	Citrus, fruity, radish	n.f.	0.01 ± 0.001	n.d.	n.d.	n.d.	0.009 ± 0
33	Aromadendrene	1437	1440	489-39-4	Wood	n.f.	0.015 ± 0.001	n.d.	n.d.	n.d.	n.d.
34	Humulene	1451	1454	6753-98-6	Woody	0.16	n.d.	0.048 ± 0.002	0.119 ± 0.025	0.057 ± 0.023	0.099 ± 0.014
35	beta-Chamigrene	1475	1476	18431-82-8	-	n.f.	0.348 ± 0.03	n.d.	0.54 ± 0.049	n.d.	0.478 ± 0.08
36	Viridiflorene	1493	1493	21747-46-6	-	n.f.	0.576 ± 0.159	n.d.	0.877 ± 0.215	0.265 ± 0.025	0.691 ± 0.08
37	alpha-Farnesene	1508	1508	502-61-4	Citrus, herbal, lavender, bergamot, myrrh, neroli, green	n.f.	0.007 ± 0.002	n.d.	n.d.	n.d.	n.d.
38	7-epi-alpha-Selinene	1515	1517	123123-37-5	-	n.f.	0.031 ± 0.017	n.d.	n.d.	0.017 ± 0.005	0.082 ± 0.028
39	Selina-3,7(11)-diene	1539	1542	6813-21-4	-	n.f.	0.015 ± 0.007	0.006 ± 0.002	n.d.	0.004 ± 0	n.d.
40	1,5-Epoxysalvial-4(14)-ene	1564	1573	88395-47-5	-	n.f.	n.d.	0.017 ± 0.002	0.05 ± 0.006	0.014 ± 0.003	0.016 ± 0.002
41	meta-Xylene	867	866	108-38-3	Plastic	1.1	n.d.	n.d.	n.d.	n.d.	0.009 ± 0.003
42	(−)-gamma-Elemene	1432	1434	29873-99-2	Green, woody, oily	n.f.	n.d.	n.d.	0.076 ± 0.045	n.d.	n.d.
43	(E)-alpha-Bergamotene	1435	1435	13474-59-4	Woody, warm, tea	n.f.	n.d.	n.d.	n.d.	0.009 ± 0.007	n.d.
44	Hexadecane	1600	1600	544-76-3	-	n.f.	n.d.	0.014 ± 0.003	0.04 ± 0.005	0.015 ± 0	0.016 ± 0.002
45	Styrene	889	893	100-42-5	Sweet, balsam, floral, plastic	0.065	n.d.	0.007 ± 0.001	0.029 ± 0.008	0.011 ± 0.002	0.012 ± 0.003
46	alpha-Guaiene	1437	1439	3691-12-1	Sweet, woody, balsam, peppery	n.f.	n.d.	n.d.	n.d.	n.d.	0.032 ± 0
47	Cadinadiene-1,4	1530	1533	16728-99-7	-	n.f.	n.d.	n.d.	0.136 ± 0.037	n.d.	n.d.
48	Amorphadiene	1458	1458	92692-39-2	-	n.f.	n.d.	0.014 ± 0	n.d.	n.d.	n.d.
49	Cubenene	1531	1532	29837-12-5	Spicy, fruity, mango	n.f.	n.d.	0.018 ± 0.012	n.d.	n.d.	0.057 ± 0.043
50	gamma-Patchoulene	1441	1441	508-55-4	-	n.f.	n.d.	n.d.	0.082 ± 0.005	n.d.	n.d.
	**Alcohols**										
51	alpha-Terpineol	1190	1189	98-55-5	Pine, terpene, lilac, citrus, woody, floral	0.086	0.011 ± 0.002	0.013 ± 0	n.d.	n.d.	n.d.
52	Cubebol	1515	1515	23445-02-5	Spicy, minty	n.f.	0.033 ± 0.022	0.042 ± 0.007	0.103 ± 0.007	0.02 ± 0.017	n.d.
53	Ledol	1563	1565	577-27-5	-	n.f.	n.d.	n.d.	n.d.	0.004 ± 0	n.d.
54	Spathulenol	1574	1576	6750-60-3	Earthy, herbal, fruity	n.f.	0.04 ± 0.004	0.013 ± 0.002	0.049 ± 0.019	0.011 ± 0.001	0.012 ± 0
55	(−)-Spathulenol	1581	1577	77171-55-2	Honey	n.f.	0.016 ± 0.004	n.d.	n.d.	n.d.	n.d.
56	Viridiflorol	1598	1591	552-02-3	Sweet, green, herbal, fruity, tropical, minty	n.f.	0.003 ± 0.001	n.d.	n.d.	n.d.	n.d.
57	Zingiberenol	1614	1616	58334-55-7	Ginger, metal	n.f.	n.d.	0.014 ± 0	0.026 ± 0.003	0.012 ± 0.003	n.d.
58	gamma-Eudesmol	1629	1631	1209-71-8	Waxy, sweet	n.f.	0.051 ± 0.028	0.024 ± 0.015	0.118 ± 0.011	n.d.	0.018 ± 0.001
59	beta-Acorenol	1649	1649	28400-11-5	-	n.f.	n.d.	n.d.	n.d.	0.094 ± 0.029	n.d.
60	alpha-Eudesmol	1651	1653	473-16-5	-	n.f.	0.085 ± 0.036	0.068 ± 0.027	0.245 ± 0.072	n.d.	0.12 ± 0.017
61	Fenchol	1112	1113	1632-73-1	Camphor, borneol, pine, woody, dry, sweet, lemon	0.78	0.009 ± 0.003	n.d.	n.d.	n.d.	n.d.
62	Junenol	1610	1617	472-07-1	-	n.f.	n.d.	0.01 ± 0.005	n.d.	0.014 ± 0.005	0.017 ± 0.001
63	beta-Eudesmol	1647	1649	473-15-4	Woody, green	n.f.	0.074 ± 0.007	0.048 ± 0.007	0.362 ± 0.335	0.066 ± 0.019	0.052 ± 0.006
64	(−)-alpha-Terpineol	1191	1190	10482-56-1	Lilac, floral, terpenic	9.18	n.d.	n.d.	0.03 ± 0.001	0.007 ± 0.003	0.006 ± 0
65	(−)-Globulol	1580	1580	489-41-8	-	n.f.	n.d.	n.d.	0.051 ± 0.008	n.d.	n.d.
66	(−)-Borneol	1164	1166	464-45-9	Pine, woody, camphor	0.08	n.d.	n.d.	0.067 ± 0.004	0.006 ± 0.001	n.d.
67	1-epi-Cubenol	1627	1627	19912-67-5	-	n.f.	n.d.	n.d.	n.d.	n.d.	0.075 ± 0.04
	**Aldehydes**										
68	Perillaldehyde	1272	1272	2111-75-3	Fresh, green, herbal, grassy, sweet, mint, cumin	0.03	0.022 ± 0.005	0.035 ± 0.014	0.11 ± 0.018	0.019 ± 0.009	0.038 ± 0.024
	**Esters**										
69	Ethyl decanoate	1398	1396	110-38-3	Sweet, waxy, fruity, apple, grape, oily, brandy	0.005	n.d.	0.006 ± 0.002	n.d.	0.009 ± 0.001	0.009 ± 0.001
70	Bornyl acetate	1285	1285	76-49-3	Woody, pine, herbal, cedar, spice	0.075	n.d.	0.013 ± 0	0.047 ± 0.032	0.009 ± 0.002	0.014 ± 0.001
71	Ethyl palmitate	1997	1993	628-97-7	Mild, waxy, fruity, creamy, milky, balsam	2	n.d.	n.d.	n.d.	0.003 ± 0	n.d.
	**Others**										
72	1-Chlorooctane	1059	1059	111-85-3	-	n.f.	0.204 ± 0.088	0.078 ± 0.005	0.345 ± 0.142	0.045 ± 0.01	0.043 ± 0.013
73	Heptadecane	1700	1700	629-78-7	-	n.f.	n.d.	0.058 ± 0.001	0.146 ± 0.028	0.055 ± 0.007	0.012 ± 0
74	2,4-Dimethyldecane	1103	1106	2801-84-5	-	n.f.	n.d.	n.d.	0.023 ± 0.011	n.d.	0.011 ± 0.001

^a^: Calculated retention index. ^b^: Matched retention index. ^c^: The odor was queried in Perflavory Search (http://www.perflavory.com/search.php; accessed on 23 August 2025) and the Good Scents Company Information System (https://www.thegoodscentscompany.com/; accessed on 23 August 2025). ^d^: Odor thresholds were obtained from VAN GEMERT L J. Odour thresholds: compilations of odour threshold values in air, water and other media [M], using detection (d) odor threshold values in mg/kg water. n.d.: not detectable; n.f.: not found; “-”: no odor description information was found in the literature; AC: hawk tea fermented with *Aspergillus cristatus*; BA: hawk tea fermented with *Blastobotrys adeninivorans*; BS: hawk tea fermented with *Bacillus subtilis*; MP: hawk tea fermented with *Monascus purpureus*; CON: hawk tea with no fermentation.

**Table 2:** The VOCs with OVAs over 1 in hawk tea samples.

Compounds	Thresholds (mg/kg)	OAVs				
		CON	BS	BA	MP	AC
(Z)-beta-Ocimene	0.034	1.494	n.d.	n.d.	n.d.	n.d.
beta-Caryophyllene	0.064	3.941	0.205	4.559	0.237	4.221
para-Cymene	0.005	n.d.	n.d.	5.566	n.d.	4.192
Perillaldehyde	0.03	0.742	n.d.	3.692	n.d.	1.278
Ethyl decanoate	0.005	40.984	n.d.	69.013	n.d.	8.624
Bornyl acetate	0.075	n.d.	1.043	1.954	0.611	0.166

n.d.: not detectable; AC: hawk tea fermented with *Aspergillus cristatus*; BA: hawk tea fermented with *Blastobotrys adeninivorans*; BS: hawk tea fermented with *Bacillus subtilis*; MP: hawk tea fermented with *Monascus purpureus*; CON: hawk tea with no fermentation.

**Table 3:** The VOCs with ROVAs over 1 in hawk tea samples.

Compounds	Thresholds (Water, mg/kg)	ROAVs				
		CON	MP	AC	BS	BA
2-Decenal	0.001	10.97	11.52	14.00	12.33	13.03
Linalool	0.00022	75.69	90.10	67.15	91.90	100.00
Decanal	0.003	4.19	3.03	2.55	2.84	2.31
Citronellal	0.006	1.36	0.77	1.50	1.46	0.77
1-Octen-3-ol	0.0015	1.10	0.90	0.95	1.06	1.04
Nonanal	0.0011	10.18	6.29	6.01	5.80	4.44
1-Hexanol	0.0056	1.02	1.02	1.02	0.97	1.44
3-Methyl-1-butanol-M	0.004	4.82	5.16	5.50	4.70	6.55
3-Methyl-1-butanol-D	0.004	1.02	1.51	1.88	1.30	2.65
1,8-Cineole	0.0011	12.77	9.06	6.96	9.43	9.51
Heptanal	0.0028	1.77	0.88	0.83	0.79	0.74
1-Butanol-D	0.4592	1.32	1.84	1.91	1.84	1.97
Diallyl sulfide-D	0.1	2.13	2.43	2.44	2.53	2.52
Hexanal-M	0.005	6.21	3.28	3.67	2.99	2.23
Hexanal-D	0.005	3.13	0.85	1.11	0.72	0.50
Butyl acetate-M	0.058	1.35	1.50	1.43	1.53	1.52
1-Penten-3-one	0.023	2.92	3.10	3.11	3.09	3.17
alpha-Pinene	0.014	1.27	1.08	1.23	1.07	1.14
Methyl 3-methylbutanoate	0.0044	0.70	0.88	1.18	0.81	0.94
Ethyl propanoate	0.01	10.72	15.80	16.11	16.05	17.07
3-Methylbutanal	0.0011	69.22	45.53	19.10	64.80	22.26
Ethyl acetate	0.005	57.75	38.41	39.56	39.05	37.82
Butanal	0.002	17.25	21.65	23.06	21.10	21.27
2-Methylpropanal	0.0015	11.05	8.52	4.55	11.62	4.10
Propanal	0.0151	6.54	3.34	3.46	3.97	2.48

AC: hawk tea fermented with *Aspergillus cristatus*; BA: hawk tea fermented with *Blastobotrys adeninivorans*; BS: hawk tea fermented with *Bacillus subtilis*; MP: hawk tea fermented with *Monascus purpureus*; CON: hawk tea with no fermentation.

**Table 4:** Sensory descriptors vocabulary created using Flash Profile method.

Attribute	Description
Appearance	Fluffy, small spots, brown
Liquor color	Clarity, yellowish brown
Aroma	Fresh scent, moldy smell, cool taste
Taste	Bitter taste, sweet taste, astringency, freshness, special taste
Leaf bottom	Leaf integrity, leaf yellowish brown, softness

## Data Availability

The original contributions presented in this study are included in the article/Supplementary Materials. Further inquiries can be directed to the corresponding author.

## Supplementary Materials

The following supporting information can be downloaded at https://www.mdpi.com/article/10.3390/foods15020324/s1: Figure S1: The chromatogram of ultra-fast GC e-nose analysis; Figure S2: The total ion chromatograms of GC-MS; Figure S3: HCA analysis of hawk tea samples with different fermentation strains based on the sensory evaluation data; Figure S4: The OPLS-DA plots comparing the CON and BA groups based on GC-MS data; Figure S5: The OPLS-DA plots comparing the CON and BA groups based on GC-IMS data; Table S1: Qualitative analysis of major differential compounds in different hawk teas by ultra-fast GC E-nose; Table S2: The VOCs identified by GC-IMS during fermentation; Table S3: Sensory descriptors created by each assessor.
