# Supplementary material for: Screening of Four Microbes for Solid-State Fermentation of Hawk Tea to Improve Its Flavor: Electronic Nose/GC-MS/GC-IMS-Guided Selection
Source: Foods. 2026 Jan 15;15(2):324. doi: 10.3390/foods15020324 (PMC12840180; doi:10.3390/foods15020324)
Supplement: Supplementary file 1 [file foods-15-00324-s001.zip › foods-4063190-supplementary.pdf]

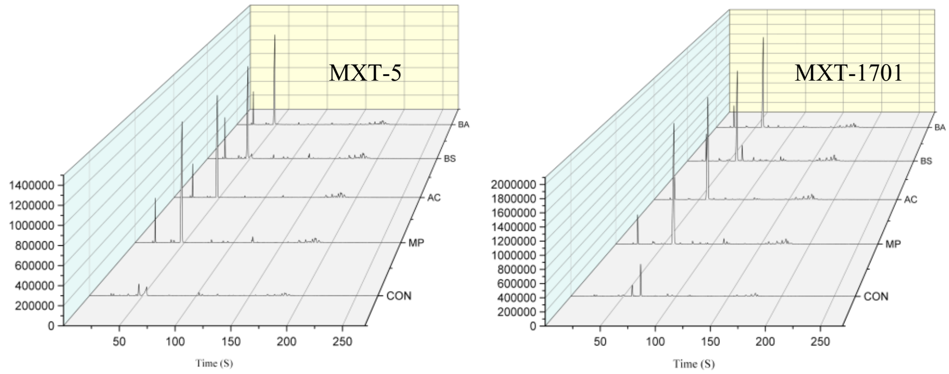

Figure S1. The chromatogram of ultra-fast GC e-nose analysis. AC group: hawk tea fermented with *Aspergillus cristatus*; BA group: hawk tea fermented with *Blastobotrys adeninivorans*; BS group: hawk tea fermented with *Bacillus subtilis*; MP group: hawk tea fermented with *Monascus purpureus*; CON group: hawk tea with no fermentation

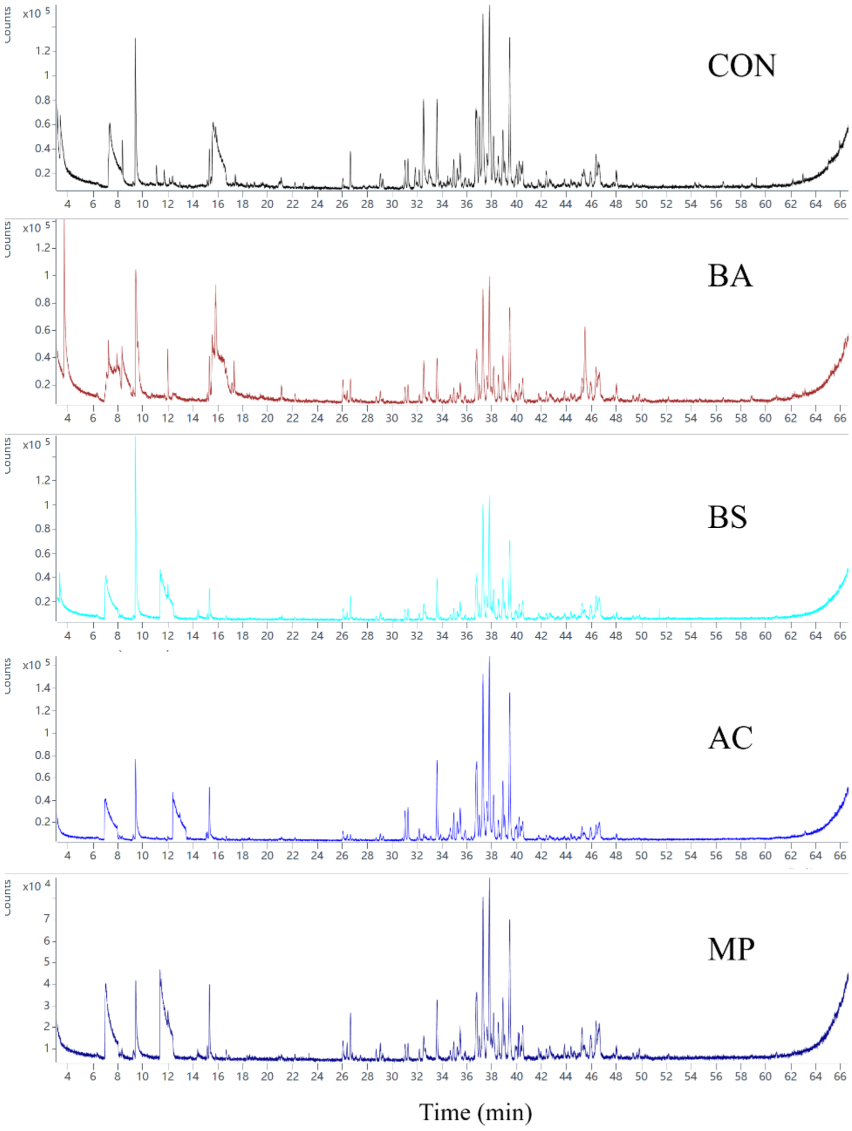

Figure S2. The total ion chromatograms of GC-MS. AC group: hawk tea fermented with

Aspergillus cristatus; BA group: hawk tea fermented with *Blastobotrys adeninivorans*; BS group: hawk tea fermented with *Bacillus subtilis*; MP group: hawk tea fermented with *Monascus purpureus*; CON group: hawk tea with no fermentation.

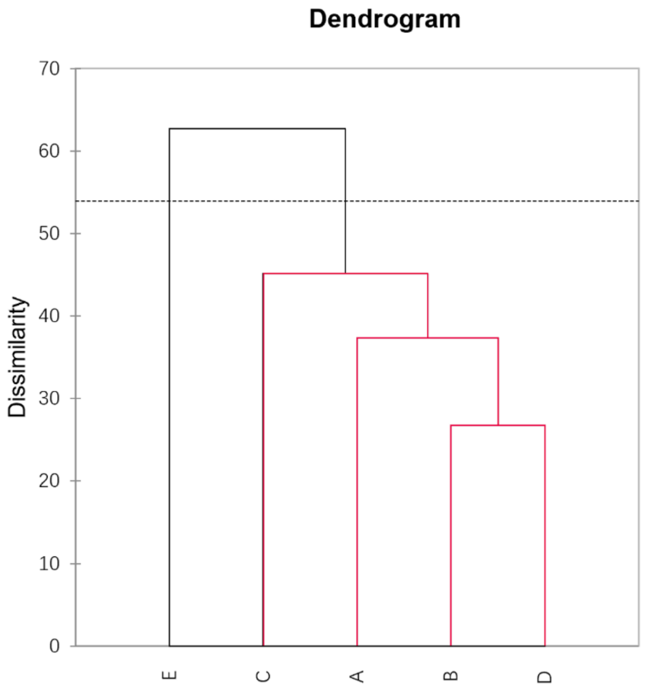

Figure S3. HCA analysis of hawk tea samples with different fermentation strains based on the sensory evaluation data. A represents BS group, B represents BA group, C represents MP group, D represents AC group, E represents CON group.

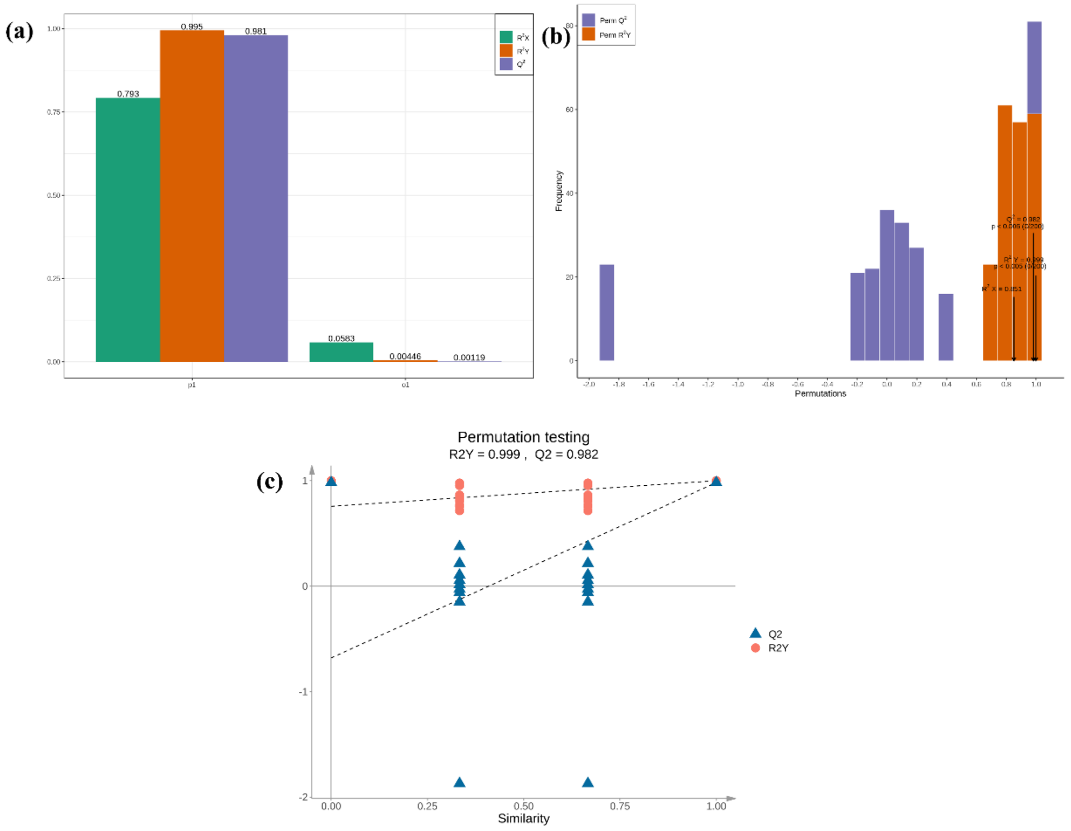

Figure S4. The OPLS-DA plot comparing the CON and BA groups based on GC-MS data. (a) OPLS-DA model overview; (b) Permutation test (200 permutations); (c) Another style polt of permutation test (200 permutations). CON group: hawk tea with no fermentation; BA group: hawk tea fermented with *Blastobotrys adeninivorans*.

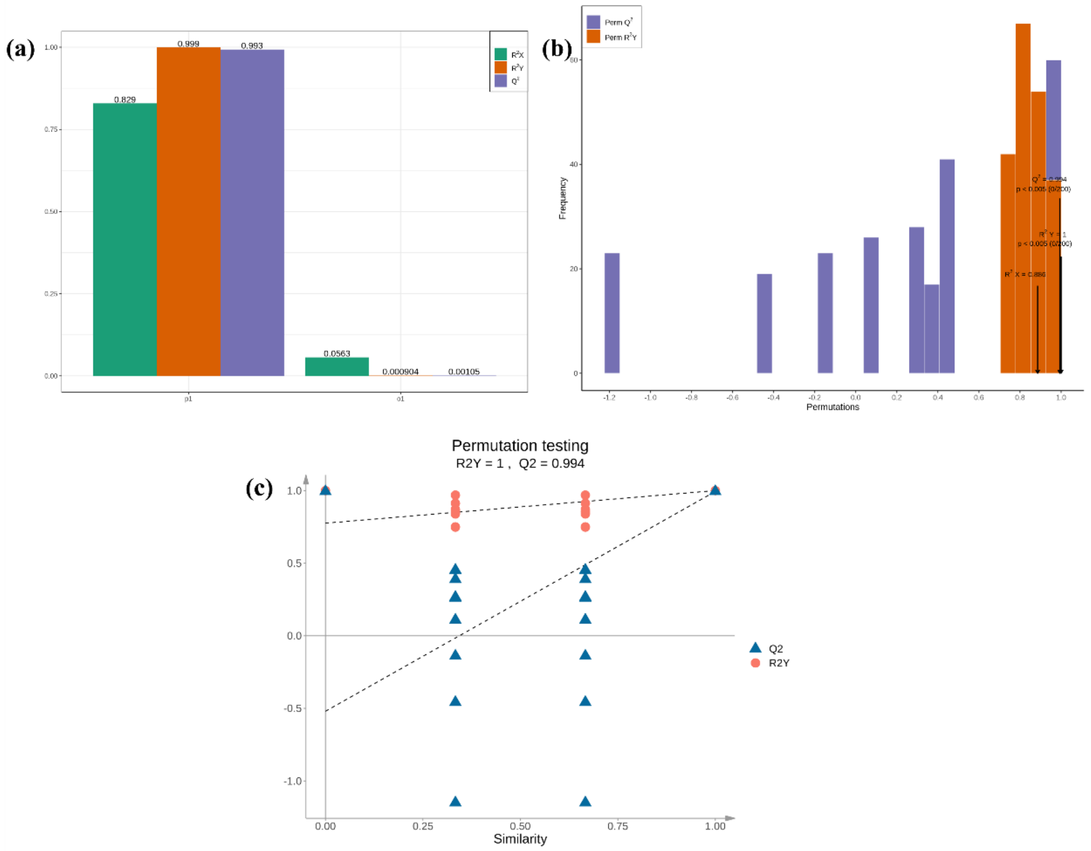

Figure S5. The OPLS-DA plot comparing the CON and BA groups based on GC-IMS data. (a) OPLS-DA model overview; (b) Permutation test (200 permutations); (c) Another style polt of permutation test (200 permutations). CON group: hawk tea with no fermentation; BA group: hawk tea fermented with *Blastobotrys adeninivorans*.

Table S1. Qualitative analysis of major differential compounds in different hawk tea by ultra-fast GC E-nose.

No.	MXT-5		MXT-1701		CAS	Compounds	Formula	Sensory Description
	t_R/s	RI	t_R/s	RI				
1	20.48	416	22.57	578	64-17-5	Ethanol	C_2H_6O	Alcoholic, ethanol, etheral, fragrant, pleasant, pungent, strong, sweet, weak
2	22.6	449	24.48	606	107-02-8	2-Propenal	C_3H_4O	Acrid, almond, cherry, choking, hot fat, pungent, sharp, sweet
3	28.45	539	30.36	644	71-23-8	1-Propanol	C_3H_8O	Alcoholic, ethanol, fermented, fruity, fusel, musty, plastic, pungent
4	32.36	599	-	-	78-93-3	2-Butanone	$C_2H_5COCH_3$	Acetone, butter, cheese, chemical, chocolate, etheral, fragrant, fruity, gaseous, pleasant, pungent, sharp, sweet
5	36.67	622	38.18	695	554-12-1	Methyl propanoate	$C_4H_8O_2$	Apple, etheral, fresh, fruity, harsh, rum, strawberry, sweet
6	39.55	637	39.36	702	108-21-4	Isopropyl acetate	$C_5H_{10}O_2$	Aromatic, banana, chemical, etheral, fruity, pleasant, sweet
7	43.97	660	49.16	748	123-73-9	But-(E)-2-enal	C_4H_6O	Floral, green, plastic, pungent

8	47.68	679	59.89	799	616-25-1	1-Penten-3-ol	$C_5H_{10}O$	Burnt, butter, fruity, grassy, green, horseradish, meaty, milky, pungent, tropical, vegetable
9	51.63	699	65.65	826	584-02-1	3-Pentanol	$C_5H_{12}O$	Fruity, green, nutty, oily, sweet
10	62.46	748	69.6	845	110-86-1	Pyridine	C_5H_5N	Amine, burnt, cold meat fat, fishy, nauseating, pungent, putrid, rancid, sharp, solvent, sour
11	68.04	774	73.04	861	556-82-1	3-Methylbut-2-en-1-ol	$C_5H_{10}O$	Fruity, green, herbaceous, lavender
12	74.79	804	72.42	858	105-54-4	Ethyl butyrate	$C_6H_{12}O_2$	Acetone, banana, bubblegum, caramelized, fruity, pineapple, strawberry, sweet
13	77.85	819	79.29	891	624-24-8	Methyl pentanoate	$C_6H_{12}O_2$	Apple, etheral, fruity, green, nutty, pineapple, sweet
14	88.15	869	85.56	923	100-41-4	Ethylbenzene	C_8H_{10}	Aromatic, etheral, floral, gasoline, pungent, sweet
15	89.75	877	87.2	931	108-38-3	m-Xylene	C_8H_{10}	Aromatic, cold meat fat, plastic, sweet

16	94.23	898	94.64	970	106-35-4	3-Heptanone	$C_7H_{14}O$	Cinnamon, fatty, fruity, green, spicy, sweet
17	102.65	943	91.9	956	80-56-8	alpha-Pinene	$C_{10}H_{16}$	Camphor, citrus, earthy, fresh, fruity, green, lime, pine, sweet, terpenic, turpentine, woody
18	105.72	959	96.24	979	5911-04-6	Nonane, 3-methyl	$C_{10}H_{22}$	-
19	118.91	1033	114.62	1083	470-82-6	1, 8-Cineole	$C_{10}H_{18}O$	Camphor, herbaceous, licorice, medicinal, mentholic, minty, pine, sweet
20	119.76	1038	111.47	1064	555-10-2	beta-Phellandrene	$C_{10}H_{16}$	Fruity, herbaceous, minty, pleasant, terpenic, turpentine
21	124.65	1067	116.92	1096	99-85-4	gamma-Terpinene	$C_{10}H_{16}$	Citrus, etheral, fruity, gasoline, herbaceous, lemon, oily, sweet, terpenic, turpentine, woody
22	131.07	1107	132.64	1200	78-70-6	Linalool	$C_{10}H_{18}O$	Anise, bergamot, citrus, floral, fragrant, fresh, fruity, green, lavender, lemon, lily, Muscat, oil, parsley, rose, spicy, sweet, terpenic, woody
23	141.68	1185	146	1308	98-55-5	alpha-Terpineol	$C_{10}H_{18}O$	Anise, citrus, floral, fruity, lilac, minty, oily, peach, pine, toothpaste, woody

24	155.61	1303	154.8	1389	112-12-9	Undecan-2-one	$C_{11}H_{22}O$	Creamy, dusty, fatty, floral, fresh, fruity, green, ketonic, musty, orange, orris, rose, strong, tallowy, waxy
25	166.71	1399	157.83	1416	6378-65-0	N-hexyl-hexanoate	$C_{12}H_{24}O_2$	Apple, fresh, fruity, green, peach, prune, vegetable
26	173.55	1451	166.28	1491	3853-83-6	alpha-Himachalene	$C_{15}H_{24}$	-
27	178.21	1487	169.42	1517	87-44-5	beta-Caryophyllene	$C_{15}H_{24}$	Fruity, green, musty, spicy, sweet, terpenic, woody
28	180.43	1503	171.48	1533	473-13-2	alpha-Selinene	$C_{15}H_{24}$	Amber, orange, pepper
29	182.97	1520	175.48	1565	10486-19-8	Tridecanal	$C_{13}H_{26}O$	Aldehydic, floral, musty, sweet
30	187.01	1546	177.34	1579	111-82-0	Methyl dodecanoate	$C_{13}H_{26}O_2$	Coconut, creamy, fatty, floral, fruity, mushroom, soapy, sweet, waxy, waxy (weak)
31	189.85	1565	186.04	1648	25152-85-6	cis-3-Hexenyl benzoate	$C_{13}H_{16}O_2$	Fatty, floral, fresh, green

Table S2. The VOCs identified by GC-IMS during fermentation (*n*=3).

No.	Compound	CAS	RI[a]	Rt[b] (s)	Dt (RIPrel)[c]	Thresholds (water, mg/kg)[d]	Odor[e]	Relative content (%)				
								CON	MP	AC	BS	BA
	**Esters**											
1	Benzyl acetate	140-11-4	1925.9	3144.296	1.31025	0.364	Sweet, jasmine floral, fruity, fresh	1.42±0.17	1.45±0.20	1.59±0.20	1.84±0.12	1.90±0.09
2	Neryl acetate	141-12-8	1796.1	2375.122	1.22808	2-8.5	Orange blossom, rose, honey, raspberry, sweet	0.70±0.05	0.72±0.05	0.71±0.05	0.82±0.21	1.33±0.17
3	Citronellyl acetate	150-84-5	1762	2206.503	1.49032	1	Lemon, fresh fruity）	1.16±0.12	1.19±0.09	1.32±0.09	1.22±0.04	1.47±0.07
4	Ethyl octanoate	106-32-1	1487.9	1220.261	1.47678	0.0193	Fruity, pineapple,	0.87±0.09	0.84±0.09	0.85±0.09	0.99±0.10	1.00±0.05

							apple, brandy				
5	Ethyl hexanoate	123-66-0	1251.3	735.826	1.34657	0.005	Pineapple, fruity, wine	0.26±0.04	0.18±0.01	0.24±0.01	0.2±0.02	0.18±0.01
6	Butyl butyrate	109-21-7	1235.5	712.585	1.3488	0.4	Fruity, banana, pineapple, green cherry, tropical fruit mature and juicy fruity aroma	0.46±0.01	0.57±0.01	0.56±0.01	0.64±0.01	0.60±0.03
7	Methyl hexanoate-M	106-70-7	1193.8	654.482	1.26616	0.07	Pineapple, apricot, fruity	3.90±0.07	4.70±0.06	4.92±0.06	4.75±0.02	4.90±0.01
8	Methyl hexanoate-D	106-70-7	1194.2	655.035	1.68494	0.07	Pineapple, apricot, fruity	1.85±0.13	2.87±0.08	3.96±0.08	2.88±0.07	3.17±0.06
9	Butyl acetate-M	123-86-4	1095.6	469.017	1.24457	0.058	Fruity	8.61±0.41	9.55±0.1	9.12±0.1	9.72±0.1	9.64±0.08

10	Butyl acetate-D	123-86-4	1096	469.547	1.61629	0.058	Fruity	8.18±1.21	8.91±0.49	6.92±0.49	9.43±0.15	8.18±0.38
11	Ethyl 3-methylbutanoate-M	108-64-5	1073.8	440.411	1.2543	0.00001	Apple, banana, sour and sweet	5.82±0.06	6.60±0.05	6.78±0.05	6.49±0.05	6.73±0.02
12	Ethyl 3-methylbutanoate-D	108-64-5	1074.2	440.941	1.65844	0.00001	Apple, banana, sour and sweet	5.30±0.28	8.02±0.32	9.96±0.32	7.67±0.17	8.14±0.15
13	Ethyl butanoate-M	105-54-4	1061	424.518	1.21324	0.0009	Pineapple, fruity, ester, whiskey	13.93±0.23	5.80±0.03	5.85±0.03	5.77±0.03	6.00±0.01
14	Ethyl butanoate-D	105-54-4	1061.9	425.578	1.58063	n.f.	Pineapple, fruity, ester, whiskey	3.00±0.17	0.83±0.02	1.23±0.02	0.77±0.01	0.93±0.01
15	Methyl 3-methylbutanoate	556-24-1	1032.7	391.184	1.52915	0.0044-0.044	Strong apple, pineapple	0.34±0.05	0.43±0.03	0.57±0.03	0.39±0.02	0.46±0.02

16	Ethyl propanoate	105-37-3	981.5	340.911	1.45178	0.01	Grape, pineapple, fruity, rum	11.75±0.48	17.32±0.21	17.66±0.21	17.60±0.09	18.71±0.17
17	Ethyl isobutyrate	97-62-1	991	348.093	1.55898	0.00002	Sweet,fruity, alcoholic, rummy	0.50±0.10	0.23±0.02	0.32±0.02	0.22±0.01	0.26±0.01
18	Ethyl acetate	141-78-6	903.3	287.418	1.33525	0.005	Fresh, fruity, sweet, grassy	31.66±0.65	21.06±0.13	21.69±0.13	21.41±0.09	20.74±0.17
19	Methyl acetate	79-20-9	858.1	260.424	1.19449	1.5-47	Ester, Green	6.91±0.19	3.09±0.09	3.30±0.09	3.03±0.04	2.86±0.14
	**Alcohols**											
20	alpha-Terpineol	98-55-5	1914.2	3065.404	1.23109	1.2	Pine terpenoid, citrus, woody, floral	5.48±0.42	2.99±0.17	2.90±0.17	8.97±0.36	5.33±0.36
21	1-Octanol	111-87-5	1672.9	1820.036	1.46054	0.1258	Citrus, sweet, herbs, waxy,	0.96±0.07	1.24±0.02	1.3±0.02	1.3±0.09	1.45±0.03

							rose, mushroom					
22	Linalool	78-70-6	1643.4	1707.561	1.2241	0.00022	Citrus, rose, woody, blueberry	1.83±0.06	2.17±0.17	1.62±0.17	2.22±0.06	2.41±0.05
23	2-Ethyl-1-hexanol	104-76-7	1549.2	1393.25	1.42574	25.4822	Citrus, fresh floral, greasy	1.04±0.08	1.00±0.05	1.17±0.05	1.12±0.08	1.12±0.01
24	Linalool oxide	60047-17-8	1463	1156.48	1.26442	n.f.	Floral	0.24±0.04	0.33±0.03	0.46±0.03	0.30±0.03	0.66±0.04
25	1-Octen-3-ol	3391-86-4	1489.8	1225.263	1.16583	0.0015	Mushroom, lavender, rose, hay	0.18±0.01	0.15±0.01	0.16±0.01	0.18±0.01	0.17±0.01
26	(Z)-3-Hexenol	928-96-1	1408.4	1027.674	1.25062	1.9	Green, herb	2.77±0.09	0.89±0.02	0.98±0.02	0.95±0.03	1.01±0.03
27	(E)-3-Hexen-1-ol	928-97-2	1387	981.283	1.2449	0.11	Moss, fresh	0.21±0.01	0.12±0.01	0.14±0.01	0.14±0.01	0.13±0.01
28	1-Hexanol	111-27-3	1375.7	957.695	1.3342	0.0056	Fresh, fruity, wine, sweet, green	0.62±0.03	0.63±0.03	0.62±0.03	0.6±0.01	0.88±0.03

29	cis-2-Penten-1-ol	1576-95-0	1343.9	894.006	0.94722	0.72	Green, plastic, rubber	1.93±0.07	0.85±0.09	0.86±0.09	0.98±0.04	1.07±0.02
30	1-Pentanol-M	71-41-0	1270.9	765.841	1.25749	0.1502	Balsamic	2.48±0.06	1.95±0.02	2.00±0.02	1.87±0.02	2.42±0.04
31	1-Pentanol-D	71-41-0	1270.9	765.841	1.51968	0.1502	Balsamic	0.35±0.02	0.29±0.01	0.30±0.01	0.27±0.02	0.43±0.01
32	3-Methyl-3-buten-1-ol	763-32-6	1253.5	739.147	1.29408	0.547125	Sweet, fruity	0.44±0.06	0.22±0.01	0.23±0.01	0.18±0.01	0.21±0.01
33	3-Methyl-1-butanol-M	123-51-3	1226.7	699.858	1.24494	0.004	Whiskey, banana, fruity	2.11±0.05	2.26±0.05	2.41±0.05	2.06±0.02	2.87±0.05
34	3-Methyl-1-butanol-D	123-51-3	1226.7	699.858	1.48951	0.004	Whiskey, banana, fruity	0.45±0.02	0.66±0.04	0.82±0.04	0.57±0.02	1.16±0
35	1,8-Cineole	470-82-6	1220	690.45	1.30748	0.0011	Camphor, refreshing herbal	1.54±0.05	1.09±0.05	0.84±0.05	1.14±0	1.15±0.01
36	1-Penten-3-ol	616-25-1	1180.4	626.813	0.94007	0.3581	Ethereal, green, tropical fruity	5.38±0.03	2.25±0.02	2.04±0.02	2.19±0.01	2.04±0.02

37	1-Butanol-M	71-36-3	1165.7	595.825	1.18352	0.4592	Wine	20.82±0.05	23.24±0.06	22.75±0.06	23.89±0.12	22.9±0.24
38	1-Butanol-D	71-36-3	1166.2	596.931	1.37895	0.4592	Wine	66.23±1.27	92.43±0.99	96.26±0.99	92.67±0.25	99.07±0.88
39	2-Methyl-1-propanol-M	78-83-1	1115	500.802	1.17758	6.5052	Fresh, alcoholic, leather	6.14±0.05	8.09±0.1	8.05±0.1	7.75±0.03	8.43±0.02
40	2-Methyl-1-propanol-D	78-83-1	1115	500.802	1.37316	6.5052	Fresh, alcoholic, leather	2.65±0.14	5.75±0.11	6.48±0.11	4.79±0.04	7.64±0.1
41	1-Propanol-M	71-23-8	1061.9	425.578	1.11274	8.5056	Alcohol, pungent	0.63±0.09	3.73±0.09	3.03±0.09	3.52±0.08	3.50±0.04
42	1-Propanol-D	71-23-8	1061.9	425.578	1.25538	8.5056	Alcohol, pungent	1.09±0.04	1.88±0.12	1.27±0.12	1.60±0.05	1.96±0.03
43	Ethanol	64-17-5	948	316.889	1.13483	950	Aromaticity	80.17±0.24	95.98±0.24	92.87±0.24	93.56±0.32	96.89±0.42
44	2-Propanol	67-63-0	941.5	312.431	1.22245	9.7879	Alcohol, spicy	6.21±0.16	7.12±0.19	10.87±0.19	5.95±0.04	9.57±0.02
45	2-Butanol	78-92-2	1045.8	406.268	1.3361	3.3	Fruity	0.75±0.02	0.54±0.01	0.70±0.01	0.68±0.03	0.58±0.03
	**Alkenes**											

46	alpha-Farnesene-M	502-61-4	1862.5	2741.34	1.43305	n.f.	Citrus, herbal, lavender, bergamot, myrrh, neroli, green	2.68±0.12	3.66±0.15	3.78±0.15	3.65±0.10	3.86±0.10
47	alpha-Farnesene-D	502-61-4	1862.9	2743.975	1.47223	n.f.	Citrus, herbal, lavender, bergamot, myrrh, neroli, green	3.31±0.28	4.10±0.10	4.20±0.10	3.92±0.20	4.62±0.11
48	(E)-beta-Farnesene	18794-84-8	1830.2	2556.914	1.45565	n.f.	Wood, citrus, sweet, herb	0.95±0.08	1.09±0.06	1.19±0.06	1.08±0.01	1.29±0.11
49	beta-Caryophyllene-M	87-44-5	1640.5	1697.013	1.43466	0.064	Sweet, woody	1.41±0.03	2.22±0	2.44±0	2.31±0.21	2.58±0.04
50	beta-Caryophyllene-D	87-44-5	1640.5	1697.013	1.51598	0.064	Sweet, woody	0.56±0.03	0.75±0.06	0.76±0.06	0.72±0.06	0.77±0.05

51	gamma-Terpinene	99-85-4	1252.8	738.04	1.21591	1	Oil, wood, terpenes, lemon, lime, herbs	0.47±0.08	0.28±0.02	0.28±0.02	0.23±0.01	0.27±0
52	Limonene-M	138-86-3	1212.5	679.936	1.21814	0.2	Lemon, sweet, orange, pine oil	0.78±0.03	1.25±0.03	1.23±0.03	1.38±0	1.49±0.05
53	Limonene-D	138-86-3	1212.9	680.49	1.3019	0.2	Lemon, sweet, orange, pine oil	0.60±0.01	0.78±0.05	0.66±0.05	0.85±0.01	0.96±0.03
54	Cumene	98-82-8	1197.5	659.462	1.13774	0.07	Strong, with slightly ragrant aromatic	0.48±0.01	0.50±0.02	0.47±0.02	0.46±0.01	0.48±0.01
55	alpha-Terpinene	99-86-5	1197.1	658.908	1.22596	0.08-0.085	Woody, lemon, citrus	0.36±0.01	0.33±0.02	0.26±0.02	0.29±0.03	0.24±0.01

56	3-Carene	13466-78-9	1141.1	547.682	1.22149	0.77	Citrus, lemon, woody	0.38±0.01	0.67±0.03	0.88±0.03	0.61±0	0.76±0.01
57	beta-Pinene	127-91-3	1123.8	516.165	1.2154	0.14	Resin, green	0.64±0.03	0.58±0.04	0.56±0.04	0.48±0.01	0.64±0.01
58	alpha-Pinene	80-56-8	1044.1	404.309	1.2122	0.014	Fresh, camphor, sweet, pine wood	1.94±0.04	1.66±0.06	1.89±0.06	1.64±0.02	1.75±0.01
	**Ethers**											
59	Diallyl sulfide-M	592-88-1	1150.9	566.496	1.12768	0.1	Garlic	10.4±0.14	8.25±0.09	7.90±0.09	8.21±0.03	7.95±0.01
60	Diallyl sulfide-D	592-88-1	1150.6	565.943	1.3287	0.1	Garlic	23.39±0.16	26.68±0.27	26.73±0.27	27.69±0.09	27.6±0.20
61	Dimethyl sulfide	75-18-3	799.7	229.221	0.95677	0.00012	Cabbage, sulfur, gasoline	15.43±0.44	1.67±0.05	2.08±0.05	1.88±0.02	1.72±0.01
	**Ketones**											
62	Acetophenone	98-86-2	1821.6	2509.49	1.18136	0.065	Sweet, spicy, almond	0.90±0.06	1.17±0.08	1.34±0.08	1.67±0.09	1.36±0.10

63	3-Methyl-2(5H)-furanone	22122-36-7	1806.8	2430.45	1.1045	n.f.	Cooked, roasted	2.62±0.20	2.51±0.08	2.46±0.08	2.60±0.19	2.56±0.08
64	gamma-Butyrolactone	96-48-0	1717.8	2005.245	1.09062	＞1	Cream, fat, caramel	1.76±0.16	0.81±0.02	0.84±0.02	1.75±0.02	0.94±0.07
65	6-Methyl-5-hepten-2-one	110-93-0	1354.4	914.449	1.17964	0.068	Citrus, fruity, mouldy, ketone	2.85±0.31	2.67±0.05	2.71±0.05	2.89±0.08	3.25±0.09
66	1-Hydroxy-2-propanone-M	116-09-6	1320.1	849.188	1.08346	10	Pungent, caramel, fresh	4.16±0.22	1.62±0.07	0.92±0.07	5.94±0.08	1.84±0.16
67	1-Hydroxy-2-propanone-D	116-09-6	1320.1	849.188	1.23345	10	Pungent, caramel, fresh	0.80±0.08	0.30±0.02	0.24±0.02	1.81±0.08	0.32±0.02
68	Cyclohexanone-M	108-94-1	1302.2	816.95	1.15559	0.28-0.67	Strong pungent, earthy	17.23±0.07	16.46±0.19	16.1±0.19	16.84±0.13	16.62±0.25

69	Cyclohexanone-D	108-94-1	1302.6	817.736	1.45786	0.28-0.67	Strong pungent, earthy	72±0.86	70.41±0.53	68.37±0.53	73.19±0.75	70.82±0.43
70	3-Penten-2-one	625-33-2	1121.1	511.397	1.09437	1.2	Fruity, turns into spicy during storage	1.25±0.04	0.69±0.01	0.69±0.01	0.66±0.01	0.69±0.02
71	1-Penten-3-one	1629-58-9	1037.3	396.442	1.09221	0.023	Strong pungent odors	7.36±0.16	7.82±0.14	7.84±0.14	7.8±0.02	8±0.04
72	4-Methyl-2-pentanone	108-10-1	1020.4	377.563	1.18983	0.24-0.64	Ketone	1.19±0.02	1.95±0.08	2.05±0.08	1.95±0.03	2.16±0.02
73	2-Pentanone	107-87-9	1007	363.199	1.37161	1.38	Acetone, fresh, sweet fruity, wine	1.90±0.22	0.65±0.04	1.15±0.04	0.63±0.02	0.73±0.02
74	2-Butanone	78-93-3	921.9	299.306	1.24203	35.4002	Fruity, camphor	5.69±0.07	5.30±0.11	4.90±0.11	5.14±0.11	5.94±0.11
75	Acetone	67-64-1	839.9	250.271	1.11525	0.832	Fresh, apple, pear	33.11±0.37	31.30±0.23	32.86±0.23	33.38±0.36	33.62±0.66
	**Aldehydes**											

76	2-Decenal	3913-71-1	1799.2	2390.93	1.47675	0.001	Sweet orange, waxy	1.20±0.06	1.26±0.11	1.53±0.11	1.35±0.03	1.43±0.08
77	Benzaldehyde	100-52-7	1557.6	1418.563	1.15614	0.75089	Bitter almond, cherry, nutty	1.71±0.02	1.39±0.06	1.41±0.06	1.51±0.04	1.29±0.05
78	Decanal	112-31-2	1540	1365.827	1.55832	0.003	Sweet, waxy, floral, citrus, aldehyde, fatty	1.38±0.06	1.00±0.18	0.84±0.18	0.93±0.1	0.76±0.07
79	Citronellal	106-23-0	1522.2	1314.056	1.35435	0.006	Lemon, lemongrass, rose	0.90±0.04	0.51±0.02	0.98±0.02	0.96±0.02	0.51±0.03
80	Methional	3268-49-3	1482.2	1205.253	1.09649	0.00045	Onion, meat, fruity	0.27±0.05	0.20±0.01	0.24±0.01	0.22±0	0.26±0.01
81	Nonanal	124-19-6	1410.9	1033.178	1.49335	0.0011	Rose, citrus, strong oily	1.23±0.08	0.76±0.05	0.72±0.05	0.70±0.02	0.54±0.01

82	2,4-Hexadienal	142-83-6	1392.2	992.291	1.11438	n.f.	Sweet, green, floral, citrus	0.20±0.02	0.12±0.01	0.10±0.01	0.16±0.01	0.13±0.01
83	(E)-2-Heptenal	18829-55-5	1337.3	881.425	1.25978	0.04	Spicy, green vegetables, fresh, fatty	0.52±0.07	0.40±0.03	0.36±0.03	0.46±0.03	0.45±0.04
84	(Z)-4-Heptenal	672-83-10	1239.7	718.672	1.13662	n.f.	Grass, oil	0.32±0.02	0.28±0.03	0.29±0.03	0.22±0.01	0.38±0.02
85	(E)-2-Hexenal-M	672-82-63	1237.4	715.352	1.18352	n.f.	Green, banana, fat	3.98±0.09	2.70±0.04	3.00±0.04	2.61±0.05	2.61±0.02
86	(E)-2-Hexenal-D	672-82-63	1237.4	715.352	1.51743	n.f.	Green, banana, fat	1.37±0.08	0.16±0.01	0.18±0.01	0.14±0.01	0.13±0.01
87	3-Methyl-2-butenal	107-86-8	1220.8	691.557	1.0953	n.f.	Fruity	0.93±0.01	0.25±0.01	0.59±0.01	0.24±0.02	0.22±0.02
88	Heptanal	111-71-7	1203.2	667.209	1.35327	0.0028	Fresh, aldehyde, fatty, green herbs, wine, fruity	0.54±0.02	0.27±0.01	0.25±0.01	0.24±0	0.23±0.01

89	Hexanal-M	66-25-1	1107.5	488.088	1.28455	0.005	Fresh, green, fat, fruity	3.40±0.14	1.80±0.03	2.01±0.03	1.64±0.06	1.22±0.02
90	Hexanal-D	66-25-1	1107.8	488.618	1.56443	0.005	Fresh, green, fat, fruity	1.72±0.06	0.47±0.01	0.61±0.01	0.40±0.03	0.28±0.01
91	Pentanal	110-62-3	1009.3	365.676	1.4294	0.012	Green grassy, faint banana, pungent	0.67±0.02	0.33±0.03	0.58±0.03	0.38±0.03	0.21±0.01
92	3-Methylbutanal	590-86-3	934.9	307.973	1.40423	0.0011	Chocolate, fat	8.35±0.03	5.49±0.39	2.3±0.39	7.82±0.18	2.69±0.11
93	Butanal	123-72-8	898.5	284.447	1.28398	0.002-0.0022	Pungent, fruity, green leaf	3.78±0.05	4.75±0.14	5.06±0.14	4.63±0.08	4.66±0.02
94	2-Methylpropanal	78-84-2	834.4	247.299	1.28025	0.0015	Banana, melon, slightly nutty	1.82±0.09	1.40±0.14	0.75±0.14	1.91±0.03	0.67±0.01

95	Propanal	123-38-6	824.7	242.098	1.14508	0.0151	Pungent, green grassy	10.83±0.16	5.54±0.30	5.72±0.30	6.57±0.20	4.11±0.07
96	(E)-2-Pentenal	1576-87-0	1155.5	575.419	1.10138	0.98	Potato, peas	0.48±0.02	0.19±0.01	0.15±0.01	0.15±0.01	0.15±0.01
	**Acids**											
97	Butanoic acid	107-92-6	1698.1	1921.818	1.16923	2.4	Strong acetic acid, cheese, butter, fruity	4.51±0.14	1.73±0.07	1.69±0.07	9.17±0.51	2.53±0.32
98	Propanoic acid	1979/9/4	1642.7	1704.906	1.12067	n.f.	Yogurt, vinegar	7.58±0.53	2.33±0.18	2.43±0.18	8.14±0.10	3.02±0.19
99	Acetic acid-M	64-19-7	1505.6	1267.783	1.05748	99	Spicy	80.68±1.66	64.13±5.10	60.84±5.10	81.10±1.00	67.75±3.71
100	Acetic acid-D	64-19-7	1506.9	1271.535	1.15283	99	Spicy	85.76±5.62	18.72±5.11	15.87±5.11	79.34±2.47	23.05±4.45
	**Unidentified**											
101	1	unidentified	1609.4	1586.686	1.29693	-	-	0.68±0.08	0.77±0.09	0.66±0.09	0.77±0.05	0.77±0.05
102	2	unidentified	1498.7	1249.024	1.45403	-	-	1.05±0.01	1.28±0.04	1.37±0.04	1.51±0.02	1.55±0.03
103	3	unidentified	1424.1	1063.057	1.26207	-	-	4.00±0.72	4.27±0.19	19.24±0.19	2.19±0.12	3.76±0.09

104	4	unidentified	1424.1	1063.057	1.54487	-	-	0.60±0.08	0.56±0.02	6.03±0.02	0.47±0.03	0.54±0.05
105	5	unidentified	1319.2	847.615	1.33306	-	-	0.89±0.01	0.34±0.02	0.19±0.02	1.59±0.03	0.43±0.05
106	6	unidentified	1235.5	712.585	1.68941	-	-	0.30±0.03	0.15±0	0.17±0	0.15±0.01	0.13±0.01
107	7	unidentified	1231.3	706.498	1.16342	-	-	0.28±0.04	0.25±0.01	0.22±0.01	0.26±0.01	0.24±0
108	8	unidentified	1220	690.45	1.59337	-	-	0.40±0.02	0.11±0.01	0.19±0.01	0.11±0	0.11±0
109	9	unidentified	1210.1	676.616	1.1556	-	-	0.35±0.03	0.24±0.02	0.21±0.02	0.25±0.02	0.22±0
110	10	unidentified	1197.1	658.908	1.0886	-	-	1.35±0.05	0.92±0.04	0.79±0.04	0.87±0.05	0.76±0.02
111	11	unidentified	1196.3	657.802	1.45824	-	-	0.56±0.02	0.67±0.02	0.68±0.02	0.64±0.04	0.63±0.01
112	12	unidentified	1137.2	540.488	1.09195	-	-	0.16±0.01	0.25±0.02	0.20±0.02	0.15±0	0.28±0.01
113	13	unidentified	1072.1	438.292	1.45313	-	-	0.78±0.07	0.63±0.03	0.53±0.03	0.71±0.04	0.54±0.04
114	14	unidentified	1062.8	426.637	1.50607	-	-	0.66±0.02	0.63±0.01	0.61±0.01	0.54±0	0.69±0.02
115	15	unidentified	1037.3	396.384	1.1805	-	-	2.13±0.02	2.79±0.01	2.86±0.01	2.88±0.04	3.4±0.07
116	16	unidentified	1034.9	393.66	1.26906	-	-	0.58±0.04	0.74±0.01	0.86±0.01	0.71±0.01	0.79±0.01
117	17	unidentified	1005.5	361.713	1.22711	-	-	1.96±0.04	1.59±0.03	1.94±0.03	1.59±0.03	1.65±0.02
118	18	unidentified	1008.8	365.18	1.30635	-	-	3.75±0.1	3.03±0.07	2.25±0.07	3.41±0.19	1.98±0.02

	**Others**											
119	2,3-Dimethylpyrazine	5910-89-4	1355.9	917.594	1.11552	0.8	Nutty	0.37±0.03	0.22±0	0.20±0	0.22±0.01	0.27±0
120	2,5-Dimethylpyrazine	123-32-0	1342.3	890.861	1.11552	1.75	Nutty, peanut, mouldy, earthy, potato, fatty, cocoa powder	0.18±0.02	0.17±0.01	0.15±0.01	0.28±0.01	0.18±0.02
121	Tetrahydrofuran	109-99-9	884.8	276.026	1.22338	n.f.	Ether	28.14±0.44	31.23±0.17	31.80±0.17	31.36±0.12	32.89±0.06

[a]: Calculated retention index; [b]: Matched retention index; [c]: The odor was queried in Perflavory Search (http://www.perflavory.com/search.php; accessed on 23 August 2025) and the Good Scents Company Information System (http://www.the goodscentscompany.com; accessed on 23 August 2025); [d]: Odor thresholds were obtained from VAN GEMERT L J. Odour thresholds: compilations of odour threshold values in air, water and other media[M], using detection (d) odor threshold values in mg/kg water; n.d.: not detectable; n.f.: not found; "-": no odor description information was found in the literature; The numbers and "*" represent unidentified peaks and formulae; AC: hawk tea fermented with Aspergillus cristatus; BA: hawk tea fermented with Blastobotrys ad-eninivorans; BS: hawk tea fermented with Bacillus subtilis; MP: hawk tea fermented with Monascus purpureus; CON: hawk tea with no fermentation.

Table S3. Sensory descriptors created by each assessor.

Attribute	Description
Appearance	Pekoe, curled, fluffy, small spots, mottled appearance, soft, sturdy and well-twisted leaves, brown, brownish-black, brown and green, reddish-brown, yellowish-brown
Liquor color	Bright, muddy, clarity, yellow, yellow-brown, reddish-brown, brilliant yellow, yellowish-brown
Aroma	Light taste, fresh scent, clean aroma, strong taste, camphor note, bitter, moldy smell, musty, lingering, mellow, grassy, minty, herbal/medicinal, cooling note, sweet, kerosene note, bitter tea note, brisk, earthy
Taste	Bitter, astringent, sweet aftertaste, fresh, tongue-numbing, musty, camphor note, brisk/refreshing, sweet, nauseating, clean aroma, kerosene note, minty, smooth, special
Leaf bottom	Broken leaves, integrity leaf, flat leaves, high tenacity, greenish, soft, slender stems, slightly curled, low tenacity, thick stems, yellow-green, intact leaves, reddish-dull, light yellow, brownish-yellow, short stems, smooth, non-brittle, brownish, reddish-brown, reddish-green, brown-black, hard, coarse
